# A streptozotocin-induced diabetic neuropathic pain model for static or dynamic mechanical allodynia and vulvodynia: validation using topical and systemic gabapentin

**DOI:** 10.1007/s00210-015-1145-y

**Published:** 2015-07-03

**Authors:** Gowhar Ali, Fazal Subhan, Muzaffar Abbas, Jehan Zeb, Muhammad Shahid, Robert D. E. Sewell

**Affiliations:** Department of Pharmacy, University of Peshawar, Peshawar, 25120 Pakistan; Fulbright Graduate Student, Department of Pharmaceutical Sciences, College of Pharmacy, South, Dakota State University, Brookings, SD 57007 USA; Cardiff School of Pharmacy and Pharmaceutical Sciences, Cardiff University, Redwood Building, King Edward VII Ave., Cardiff, CF10 3NB UK

**Keywords:** Vulvodynia model, Allodynia, Streptozotocin, Diabetic neuropathic pain, Gabapentin

## Abstract

Neuropathic vulvodynia is a state of vulval discomfort characterized by a burning sensation, diffuse pain, pruritus or rawness with an acute or chronic onset. Diabetes mellitus may cause this type of vulvar pain in several ways, so this study was conducted to evaluate streptozotocin-induced diabetes as a neuropathic pain model for vulvodynia in female rats. The presence of streptozotocin (50 mg/kg i.p.)-induced diabetes was initially verified by disclosure of pancreatic tissue degeneration, blood glucose elevation and body weight loss 5–29 days after a single treatment. Dynamic (shortened paw withdrawal latency to light brushing) and static (diminished von Frey filament threshold pressure) mechanical allodynia was then confirmed on the plantar foot surface. Subsequently, both static and dynamic vulvodynia was detected by application of the paradigm to the vulval region. Systemic gabapentin (75 mg/kg, i.p.) and topical gabapentin (10 % gel) were finally tested against allodynia and vulvodynia. Topical gabapentin and the control gel vehicle significantly increased paw withdrawal threshold in the case of the static allodynia model and also paw withdrawal latency in the model for dynamic allodynia when compared with the streptozotocin-pretreated group. Likewise, in the case of static and dynamic vulvodynia, there was a significant antivulvodynia effect of systemic and topical gabapentin treatment. These outcomes substantiate the value of this model not only for allodynia but also for vulvodynia, and this was corroborated by the findings not only with systemic but also with topical gabapentin.

**Electronic supplementary material:**

The online version of this article (doi:10.1007/s00210-015-1145-y) contains supplementary material, which is available to authorized users.

## Introduction

The prevalence of diabetes mellitus has been increasing globally, and it was estimated by the WHO that the occurrence of diabetes in adults was 173 million in 2002 (Shaw et al. 2010). Painful diabetic neuropathy is a complication of diabetes mellitus, and it is encountered in 60 % of patients adversely affecting their quality of life (Said 2007). In neuropathic pain, spontaneous and pathologically exaggerated responses occur to noxious and non-noxious stimuli (Costigan et al. 2009). Underlying mechanisms implicated in neuropathic pain include synaptic facilitation which contributes to secondary hypersensitivity and tactile allodynia (Campbell and Meyer 2006).

Vulvodynia is a state of vulval discomfort characterized by burning, diffuse moderate to severe pain, pruritus or rawness with acute or chronic onset. Usually, there are few visible symptoms, although varying degrees of erythema have been reported (Bachmann et al. 2006; Lynch 1986; Paavonen 1995; Young et al. 1984), and the most frequently identified types are generalized unprovoked vulvodynia or vestibulodynia (Backonja et al. 1998). Diabetes mellitus may cause vulvar pain of this type in several ways (Kalra et al. 2013), and currently, the management of neuropathic pain involves adjunctive therapy. It is a challenge to define an exact course of treatment for instance in diabetic neuropathy since active pain mechanisms in patients are still not clearly defined and often expressed as complex pain phenotypes. Consequently, only partial relief is achievable in most individuals (Goodnick et al. 2000; Field et al. 1999). It has been shown that diabetic and para-diabetic neuropathic pain is invariably resistant to classical non-steroidal anti-inflammatory drugs (NSAIDs) and opioids or that it has a poor response to these agents (James and Page 1994).

In this connection, numerous drugs have been extensively investigated against painful diabetic neuropathy including gabapentin (Gorson et al. 1999; Field et al. 1999; Zhang et al. 2013). Moreover, analgesic antidepressants have also been shown to promote the responsiveness of neurons in the locus coeruleus to noxious stimulation in a chronic neuropathy model (Alba-Delgado et al. 2012) though gabapentin has not been studied in this respect. An earlier retrospective clinical study concluded that topical gabapentin may be useful in neuropathic pain including allodynia in the area of the vulva (vulvodynia) (Boardman et al. 2008). Gabapentin [1-(aminomethyl) cyclohexane acetic acid] is an antiepileptic drug and structural analogue of the neurotransmitter gamma-aminobutyric acid (GABA). It was introduced in 1993 by the US FDA as an adjuvant anticonvulsant (Barrueto et al. 2002) and subsequently for various chronic pain conditions including diabetic neuropathy (Apkarian et al. 2009; Bennett and Simpson 2004; Gilron 2007; Gilron et al. 2005; Hunter et al. 1997; Mao and Chen 2000; Wiffen et al. 2005; Leo 2013). In this context, analgesic effects on peripheral nociception in rats have been reported to injectable gabapentin (Carlton and Zhou 1998; Cesena and Calcutt 1999).

The primary aim of the study was to employ a streptozotocin-induced neuropathic pain model to establish the existence of mechanical allodynia and then to investigate the presence of any potential static or dynamic mechanical vulvodynia. Subsequently, the objective was to evaluate the activity of gabapentin either administered systemically or applied locally as a topical gel preparation against vulvodynia as a mode of further validating the model for predicting relevance to the clinic (Bates and Timmins 2002).

## Methods and materials

### Chemicals and instruments

A von Frey filament (hair) kit was obtained from Stoelting (USA). The following were purchased from suppliers: streptozotocin (Sigma-Aldrich), glucometer (Roche Diagnostic Corporation, Germany), blood glucose strips (Optium Xceed, Abbott), gabapentin gel 10 % *w*/*w* and the control gel base (a liposome-containing oil in water gel comprising xanthan gum hydrocolloid with polyacrylamide) minus the active pharmaceutical ingredient [API]) were supplied by St Mary’s Pharmaceutical Unit (SMPU, Cardiff, UK) under their Manufacturer’s Special License (MS). Gabapentin was obtained from MKB Pharmaceuticals (Pvt.) Ltd., Peshawar, Pakistan. All other chemicals and reagents used were of analytical grade.

### Animals

Sprague-Dawley female rats, bred in the animal house and bioassay laboratories of the Department of Pharmacy, University of Peshawar, were used throughout experimental studies. Animals were housed in transparent cages with free access to standard laboratory food and water available ad libitum. All experimental procedures were carried out between 0800 and 1700 h. A 12–12-h light and dark cycle was provided with an ambient temperature maintained at 22.0 ± 2.0 °C through a reversible air conditioning system.

### Ethical approval

The study of the topical gabapentin application was approved under a project entitled ‘Studies on the effects of topical formulations of different analgesics on allodynia and vulvodynia components of diabetic neuropathic pain’ by the Ethical Committee of the Department of Pharmacy, University of Peshawar, Peshawar, Khyber Pakhtunkhwa (KP), Pakistan, who issued approval certificate no 13/EC-12/Pharm. The study of systemic gabapentin administration was approved under a project entitled ‘Evaluation of the anti-inflammatory, antinociceptive and antipyretic activities of selected synthetic and natural compounds in animal models’ (approval certificate no 15/EC/Pharm). Moreover, all animal procedures were conducted strictly according to the Animals Scientific Procedure Act (1986) of UK.

### Induction of diabetes

Female Sprague-Dawley rats, weighing 175–200 g (age range = 7.0 ± 1.0 weeks), were randomly divided into saline vehicle and treatment groups. Sixteen hours after fasting (Babu et al. 2003), animals were weighed and a single intraperitoneal (i.p.) injection of streptozotocin (STZ) (50 mg/kg) was administered for the induction of diabetes (Field et al. 1999). Since streptozotocin has a stability problem (Rakieten et al. 1963), fresh solutions were prepared for each period of administration. Sixty minutes after streptozotocin administration, animals were allowed free access to food and water and then kept under close observation for the next 5 days during the development of diabetic symptoms. To maintain cleanliness and avoid development of any infection due to excessive urination, animal bedding was changed frequently. The singly administered dose of streptozotocin in our study was lower than that employed by other research groups in rats (Indolfi et al. 2001; Wei et al. 2003) and was in point of fact only half of that given by Indolfi et al. (2001). Additionally, at the 50 mg/kg streptozotocin dose level, no undue adverse behavioural effects have been previously reported (Field et al. 1999; Jolivalt et al. 2006). Consequently, the degree of insulinopoenia in this study was not supplemented by exogenous insulin administration, and this has been corroborated by chronological survival rates even up to 6-fold longer than our study duration (Wei et al. 2003).

### Measurement of blood glucose and body weight

Blood glucose levels of animals were measured at predetermined time intervals, i.e. experimental days 0, 5, 15 and 29 using a glucometer (Roche Diagnostic Corporation, Germany). Blood samples were collected from the rats by the tail tip method. Body weights of the diabetic and saline-treated animals were also recorded throughout the experimental protocol.

### Assessment of static allodynia in diabetic female rats

#### Pretreatment schedule

On the 29th day post-streptozotocin administration, animals were acclimatized to cages with wire mesh bottoms for 15–45 min. A series of von Frey filaments (0.4, 0.6, 1, 1.4, 2, 4, 6, 8, 10 and 15 g) were applied perpendicularly to the mid-plantar surface of the right hind paw to an extent that caused the hairs to bend (Calcutt and Chaplan 2009). Each von Frey filament was applied for a period of up to 6 s as a cut-off time or until an escape response (paw withdrawal threshold (PWT) or licking) occurred. The withdrawal threshold was defined as the minimum applied force (*g* ± SD) required to induce a paw withdrawal reflex (Field et al. 1999). Lifting of the paw or licking was recorded as a positive response, and a succeeding von Frey filament of lower force was applied for the following recording. However, in the case of an absence of response, the subsequent von Frey filament of higher force was applied. This procedure was continued until four measurements were taken after an initial change including the first one, in response (positive response) or five consecutive negative responses (2, 4, 6, 8 and 10-*g* force) or four consecutive positive responses (1.4, 1, 0.6 and 0.4-*g* force). A force of 15 *g* was selected as the cut-off force at which point further application ceased. von Frey filaments were applied at intervals of several seconds in order to avoid any influence of previous stimuli on behaviour. Any ambulation was noted as an indefinite response, and the stimulus was repeated. Static allodynia was indicated by a reduced force required to induce a paw withdrawal reflex.

#### Post-treatment schedule

Animals included for the post-drug-treatment study were those which exhibited a mean response of ≤3.63 *g* (see Table 1) (Field et al. 1999). In the topical gabapentin investigation, a uniform amount of 10 % gabapentin gel (1.0 mL/cm^2^) was applied topically for three times daily (0800, 1200 and 1600 h) on the plantar surface of the right hind paw. Static allodynia was measured 1 h post-drug application. The concentration of gabapentin applied in the form of gel (i.e. 100 mg/cm^2^) was 3.3-fold higher than that previously employed for topical lidocaine in hypoalgesic studies on mucous membranes (Schønemann et al. 1992). Control animals received an equivalent application of control gel (1.0 mL/cm^2^). In the case of the systemic study, gabapentin (75 mg/kg, i.p.) was administered to animals meeting the selection criterion, and static allodynia was measured at 1 and 2 h post-drug administration. Control animals received an equivalent amount of normal saline vehicle. The von Frey PWT inclusion criterion for static vulvodynia is shown in Table 1. The overall mean PWT values were calculated from the pooled post-treatment triplicate daytime readings (0800, 1200 and 1600 h) of static allodynia in the topical study or the duplicate readings (1 and 2 h) post-administration in the systemic study.

**Table 1:** Inclusion criteria for static and dynamic allodynia or vulvodynia

Static allodynia	Dynamic allodynia	Static vulvodynia	Dynamic vulvodynia
≤3.63-*g* von Frey paw withdrawal threshold (PWT)	≤8.0-s brushing paw withdrawal latency (PWL)	≤0.16-*g* von Frey flinching response threshold (FRT)	≤4.0-s brushing flinching response latency (FRL)

### Assessment of dynamic allodynia in diabetic female rats

On the 29th day post-streptozotocin injection, dynamic allodynia was assessed by lightly brushing the plantar surface of the hind paw of each animal with a cotton bud. The time to flinching or licking of the paw was regarded as the paw withdrawal latency (PWL) in seconds. Animals responding to the cotton bud within 8 s were selected for the study (Field et al. 1999). Fifteen seconds was selected as cut-off time beyond which the assessment was terminated.

In the topical study, gabapentin gel (10 %; 100 mg/cm^2^) or control gel (1.0 mL/cm^2^) was then applied to the hind paw, three times daily (0800, 1200 and 16.00 h), and animals were tested for dynamic allodynia, by the method described above, 1 h post-gabapentin gel or control gel application. Systemic gabapentin (75 mg/kg, i.p) was administered and dynamic allodynia was measured by the method described above at 1 and 2 h post-drug administration. The brushing PWL inclusion criterion for dynamic allodynia is shown in Table 1. The overall mean PWL values were calculated from the pooled post-treatment triplicate daytime readings (08.00, 12.00 and 16.00 h) of dynamic allodynia in the topical study or the duplicate readings (1 and 2 h) post-administration in the systemic study.

### Assessment of static vulvodynia in diabetic female rats

#### Pretreatment schedule

The anogenital area including the mons pubis area of female rats was carefully shaved. The animals were then acclimatized to cages with wire mesh bottoms for 15–45 min (to prevent excessive exploration and major grooming activities). A series of von Frey filaments (0.008, 0.02, 0.04, 0.07, 0.16, 0.4, 0.6 and 1.0 g) were applied perpendicularly for a period of 4 s and the flinching response threshold (FRT, *g*) recorded. This procedure was continued for up to four measurements after an initial change in response (positive response) or five consecutive negative responses (0.07, 0.16, 0.4, 0.6 and 1-*g* force) or two consecutive positive responses (0.008 and 0.02-*g* force). Ambulation was noted as an ambiguous response, and if it occurred, the stimulus was repeated. A von Frey force of 1 *g* was selected as the cut-off beyond which the assessment was terminated.

#### Post-treatment schedule

On the 29th day post-streptozotocin administration, a standardized testing paradigm (Chaplan et al. 1994) was used with modifications for the vulvodynia study. The von Frey FRT inclusion criterion for static vulvodynia is shown in Table 1.

Gabapentin gel (10 %; 100 mg/cm^2^) was then applied topically for three times daily (0800, 1200 and 1600 h) to the anogenital region. Static vulvodynia was assessed 1 h after gabapentin gel application. Control gel (1.0 mL/cm^2^) was applied in the control animal group. Systemic gabapentin (75 mg/kg, i.p) was administered, and static vulvodynia was measured by the method described above at 1 and 2 h post-drug administration, the controls receiving an equivalent amount of i.p. normal saline. The overall mean FRT values were calculated from the pooled post-treatment triplicate daytime readings (800, 1200 and 1600 h) of static vulvodynia in the topical study or the duplicate readings (1 and 2 h) post-administration in the systemic study.

### Assessment of dynamic vulvodynia in diabetic rats

The anogenital area of animals was shaved as before. The animals were then acclimatized on wire mesh cages for 15–45 min. On the 29th day post-streptozotocin administration, dynamic vulvodynia was assessed by lightly brushing the anogenital region for a period of 10 s or until a flinching response occurred (flinching response latency (FRL)). If no response occurred within 10 s (cut-off time), then the procedure was terminated and the animal was excluded from the study. The brushing FRL inclusion criterion for dynamic vulvodynia is shown in Table 1.

Animals meeting the inclusion criteria in the topical study had gabapentin gel (10 %; 100 mg/cm^2^) or control gel (1.0 mL/cm^2^) applied for three times daily (0800, 1200 and 1600 h) and were tested for dynamic vulvodynia 1 h later. Systemic gabapentin (75 mg/kg, i.p) was administered, and animals were tested for dynamic vulvodynia by the method described above at 1 and 2 h post-drug administration, the controls receiving an equivalent amount of i.p. normal saline. The overall mean FRL values were calculated from the pooled post-treatment triplicate daytime readings (0800, 1200 and 1600 h) of dynamic vulvodynia in the topical study or the duplicate readings (1 and 2 h) post-administration in the systemic study.

### Microscopic examination of the pancreas

**Fig. 1:**
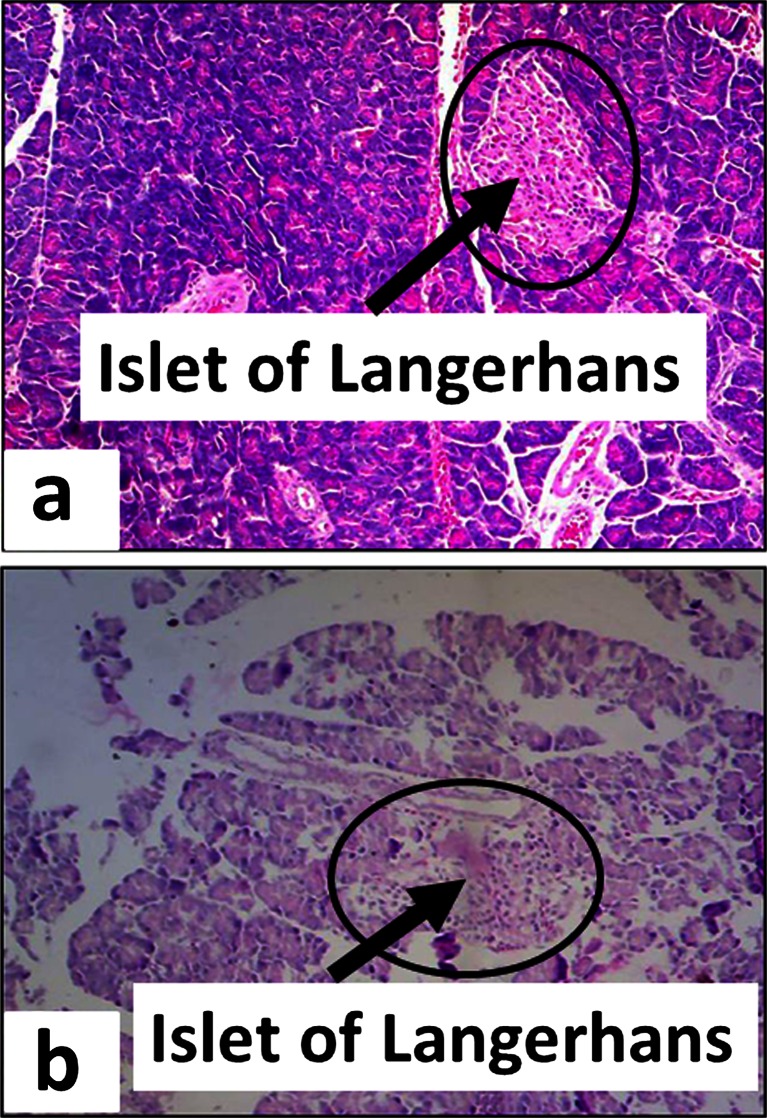
Photomicrographs of the pancreas from rats treated with **a** saline or **b** streptozotocin (50 mg/kg) i.p. showing intact or degenerated islets of Langerhans, respectively, 29 days after treatment

The pancreas was removed from each animal at the end of the experiment and stored in 10 % formalin solution for microscopic examination of islet of Langerhans cells. The tissues were then processed and slides of 5-μm thickness were prepared employing routine haematoxylin and eosin staining procedures (Falkeholm et al. 2001) (Fig. 1).

### Statistical analysis

Data were expressed as means and were analyzed by one-way ANOVA and Tukey’s multiple comparison post hoc test. Mann-Whitney *U* test was applied to data where applicable. Values were considered significant at *P* < 0.05.

## Results

### Development of diabetes in streptozotocin-pretreated animals

**Fig. 2:**
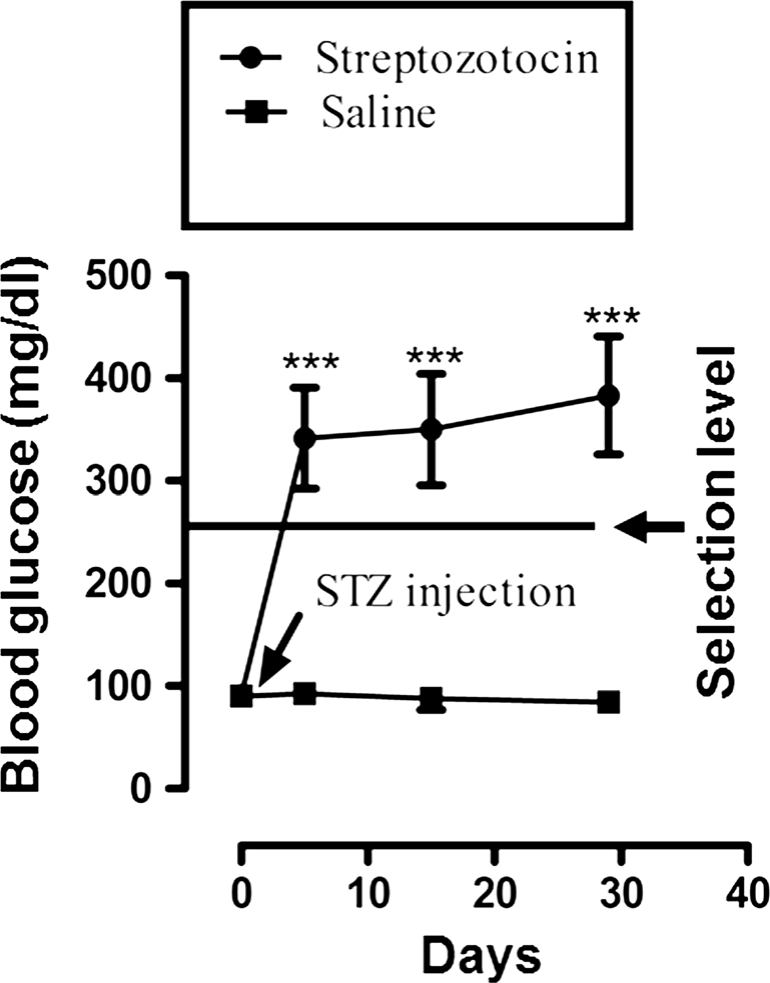
The effect of streptozotocin (STZ, 50 mg/kg i.p.) or saline vehicle on blood glucose levels of rats (mean ± SEM) measured on days 0, 5, 15 and 29 of the experimental protocol. Values for streptozotocin-treated animals were significantly different compared to saline-vehicle-treated controls (***P < 0.001, Mann–Whitney *U* test). The animal selection level for subsequent allodynia studies was ≥270 mg/dL on day 5

**Fig. 3:**
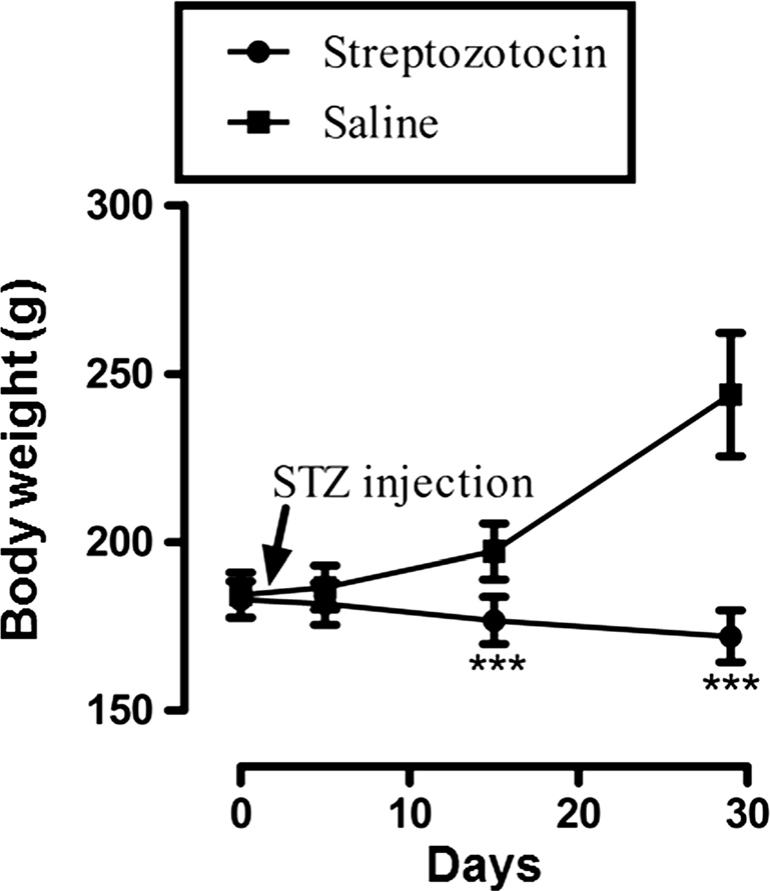
Effect of streptozotocin (STZ, 50 mg/kg i.p.) or saline vehicle on rat body weights (mean ± SEM) measured on days 0, 5, 15 and 29 of the experimental protocol. Values for streptozotocin-treated animals were significantly different compared to saline-vehicle-treated controls (***P < 0.001, Mann–Whitney *U* test)

As disclosed in Fig. 2, animals administered streptozotocin manifested diabetic symptoms reproducibly by increasing the group mean blood glucose level from 91.0 ± 1.7 mg/dL on day 0 to 342.6 ± 8.0 mg/dL on the fifth day, 350.1 ± 8.8 mg/dL on day 15, and 383.3 ± 9.3 mg/dL on day 29 of the experimental schedule. However, at a streptozotocin dose of 50 mg/kg (Field et al. 1999), animals exhibiting mean blood glucose levels ≥270 mg/dL on day 5 were selected for the study (Jolivalt et al. 2006). Development of diabetes was further confirmed from the degradation of pancreatic islet cells in streptozotocin-pretreated diabetic rats (Fig. 1). In addition to hyperglycaemia, diabetic animals exhibited mild polyphagia, polydipsia and polyuria and some body weight loss (Fig. 3).

### Activity of topical gabapentin gel (10 %) and systemic gabapentin (75 mg/kg i.p.) on static allodynia in female diabetic rats

**Fig. 4:**
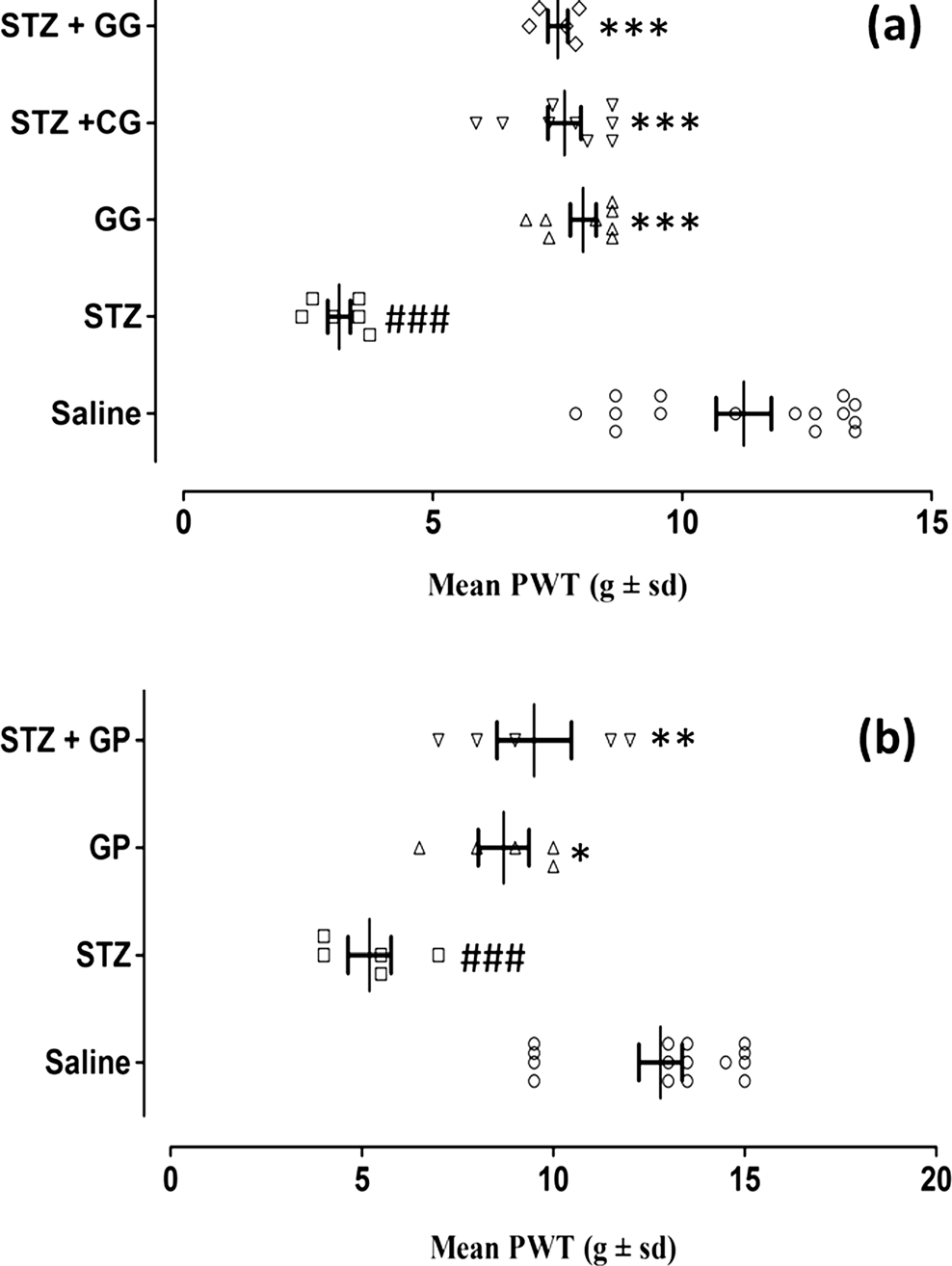
The static antiallodynia activity of topical gabapentin gel (10 %) and systemic gabapentin (75 mg/kg i.p.) in streptozotocin-induced female diabetic rats. Scatter plots showing mean paw withdrawal thresholds (PWTs, *g* ± SD) in response to von Frey filaments were determined in all groups. **a** Topical gabapentin gel (10 %) alone without streptozotocin pretreatment (*triangle*, GG, *n* = 8), streptozotocin pretreatment followed by control gel (*inverted triangle*, STZ + CG, *n* = 9) or streptozotocin pretreatment followed by gabapentin gel (*diamond*, STZ + GG, *n* = 5) was applied on the plantar surface of the right hind paws of rats for three times daily and PWT was measured 1 h after application. Significance of differences in PWT is shown between topical saline (*white circle*, *n* = 15) and streptozotocin pretreatment (*white square*, STZ, *n* = 6; ###*P* < 0.001) and between streptozotocin pretreatment (*white square*, STZ, *n* = 6) versus gabapentin gel alone (*triangle*, GG, *n* = 8; ***P < 0.001), streptozotocin pretreatment with control gel (*inverted triangle*, STZ + CG, *n* = 9; ***P < 0.001) and streptozotocin pretreatment plus gabapentin gel (*diamond*, STZ + GG, *n* = 5; ***P < 0.001), ANOVA followed by Tukey’s post hoc test. **b** In the case of the systemic study, gabapentin (75 mg/kg) was administered intraperitoneally (i.p.) and PWT was measured using the same protocol as gabapentin gel. ANOVA followed by Tukey’s post hoc test revealed statistical differences in PWT between saline (*white circle*, *n* = 15) and streptozotocin pretreatment (*white square*, STZ, *n* = 6; ###*P* < 0.001), between streptozotocin pretreatment (*white square*, STZ, *n* = 6) and gabapentin alone (*triangle*, GP 75 mg/kg, *n* = 5; *P < 0.05) and also between streptozotocin plus gabapentin (*inverted triangle*, STZ + GP 75 mg/kg, *n* = 8; **P < 0.01)

The pooled mean von Frey filament pressures (*g* ± SD) calculated as PWT values (static allodynia) are plotted in Fig. 4. In the topical application experiments (Fig. 4a), the treatment groups consisted of saline, gabapentin gel alone (GG), streptozotocin pretreatment alone (STZ), and streptozotocin pretreatment plus either topical gabapentin gel (STZ + GG 10 %) or topical control gel (STZ + CG) (*F*_(4,31)_ = 36.41, *P* < 0.001). Tukey’s post hoc analysis revealed a significant decrease (*P* < 0.001) in the PWT responses of streptozotocin-pretreated animals versus saline-treated controls, and this was reversed in the group pretreated with streptozotocin and then treated with topical gabapentin gel (STZ + GG, *P* < 0.001). There were also increased PWT values for both gabapentin gel (*P* < 0.001) and control gel vehicle (*P* < 0.001) topical treatments compared to the streptozotocin-pretreated control (STZ) group (Fig. 4a).

In the systemic study, the mean pooled von Frey filament pressures (*g*) calculated as PWT values (static allodynia) were graphed. The four groups consisted of saline controls, gabapentin alone (GP 75 mg/kg i.p), streptozotocin pretreatment alone (STZ) and streptozotocin plus gabapentin (STZ + GP (75 mg/kg i.p.) (*F*_(3,16)_ = 15.92, *P* < 0.001) (Fig. 4b). Tukey’s post hoc analysis test revealed a decrease (*P* < 0.001) in the PWT response to streptozotocin pretreatment versus saline-treated controls. A reversal of this streptozotocin-decreased PWT occurred when combined with systemic gabapentin (STZ + GP, *P* < 0.01) at the pooled 1 and 2-h post-treatment times, whereas gabapentin alone induced a response that was greater than streptozotocin alone (*P* < 0.05) (Fig. 4b).

### Activity of topical gabapentin gel (10 %) and systemic gabapentin (75 mg /kg i.p) on dynamic allodynia in female diabetic rats

**Fig. 5:**
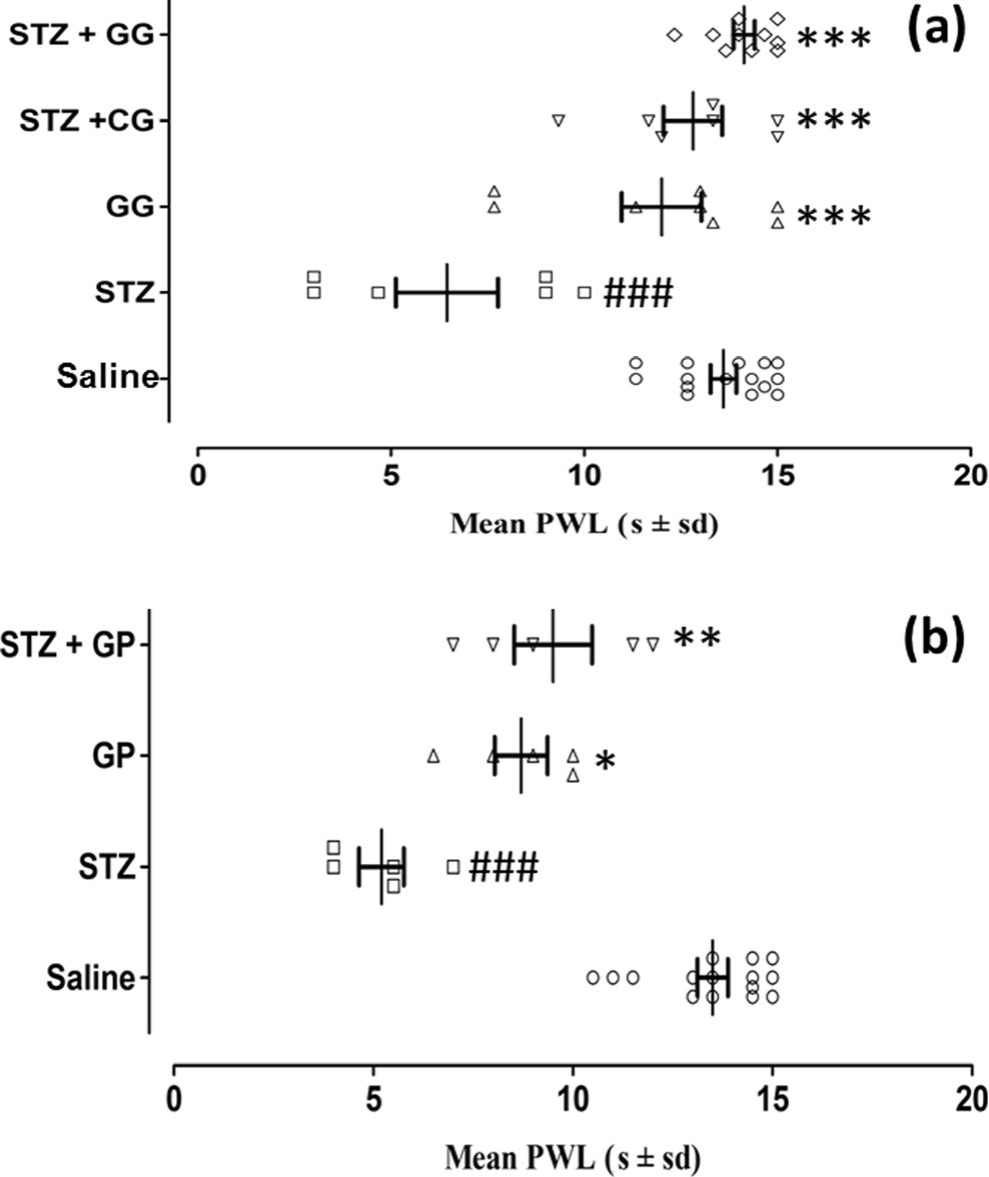
The dynamic antiallodynia activity of gabapentin gel 10 % and systemic gabapentin (75 mg/kg i.p.) in streptozotocin-induced female diabetic rats. Scatter plots showing mean paw withdrawal latency (PWL, s ± SD) in response to light brushing were determined in all groups (*n* = animal group numbers meeting the inclusion criteria are shown in *brackets*). **a** Topical control gabapentin gel alone without streptozotocin treatment (*triangle*, GG, *n* = 8), streptozotocin pretreatment followed by gabapentin gel (10 %) (*diamond*, STZ + GG, *n* = 10) or streptozotocin pretreatment followed by control gel (*inverted triangle*, STZ + CG, *n* = 7) was applied on the plantar surface of the right hind paw of rats for three times daily and PWL was measured 1 h after application. Significance of differences in PWL is shown between topical saline (*white circle*, *n* = 15) and streptozotocin pretreatment (*white square*, STZ, *n* = 6, ###*P* < 0.001) and between streptozotocin pretreatment (*white square*, STZ, *n* = 6) versus gabapentin gel alone (*triangle*, GG, *n* = 8, ***P < 0.001), streptozotocin treatment followed by control gel, i.e. STZ + CG (*inverted triangle*, *n* = 7; ***P < 0.001) and streptozotocin treatment followed by gabapentin gel, i.e. STZ + GG (10 %) (*diamond*, *n* = 10; ***P < 0.001), ANOVA followed by Tukey’s post hoc test. **b** In the case of the systemic study, the same protocol as topical gabapentin was used but gabapentin (*triangle*, GP, 75 mg/kg) was administered i.p. and PWL was measured. Statistical significance of differences in PWL is shown between saline (*white circle*, *n* = 15) and streptozotocin control (*white square*, STZ, *n* = 6) (###*P* < 0.001) and between streptozotocin control (*white square*, STZ, *n* = 6) and gabapentin alone (*triangle*, GP, *n* = 8; *P < 0.05) and STZ + GP 75 mg/kg (*inverted triangle*, *n* = 5; **P < 0.01)

Dynamic allodynia was assessed by lightly brushing a cotton bud over the plantar surface of the hind paw of rats, and the pooled PWL (mean s ± SD) was determined (*F*_(4,34)_ = 13.99, *P* < 0.001) and plotted as shown in Fig. 5a. There was a significant decrease in PWL caused by streptozotocin pretreatment in comparison with the saline-treated controls (*P* < 0.001). This reduced PWL induced by streptozotocin was totally reversed by combination with topical gabapentin gel (STZ + GG, *P* < 0.001) and also somewhat unpredictably by the control gel (STZ + CG, *P* < 0.001).

In the systemic study, dynamic allodynia was assessed using the same protocol. PWL (mean s ± SD) with respect to time after treatment at the grouped 1 and 2-h intervals (*F*_(3,16)_ = 15.92, *P* < 0.001) is depicted in Fig. 5b. Streptozotocin pretreatment reduced the PWL compared to saline treatment (*P* < 0.001), and this was reversed by combination with systemic gabapentin administration (STZ + GP, *P* < 0.01). However, as might be expected, there was also a statistical difference between the group treated with gabapentin alone (GP) compared with those pretreated with streptozotocin (*P* < 0.05, Fig. 5b).

### Activity of topical gabapentin gel (10 %) and systemic gabapentin (75 mg/kg i.p) on static vulvodynia in female diabetic rats

**Fig. 6:**
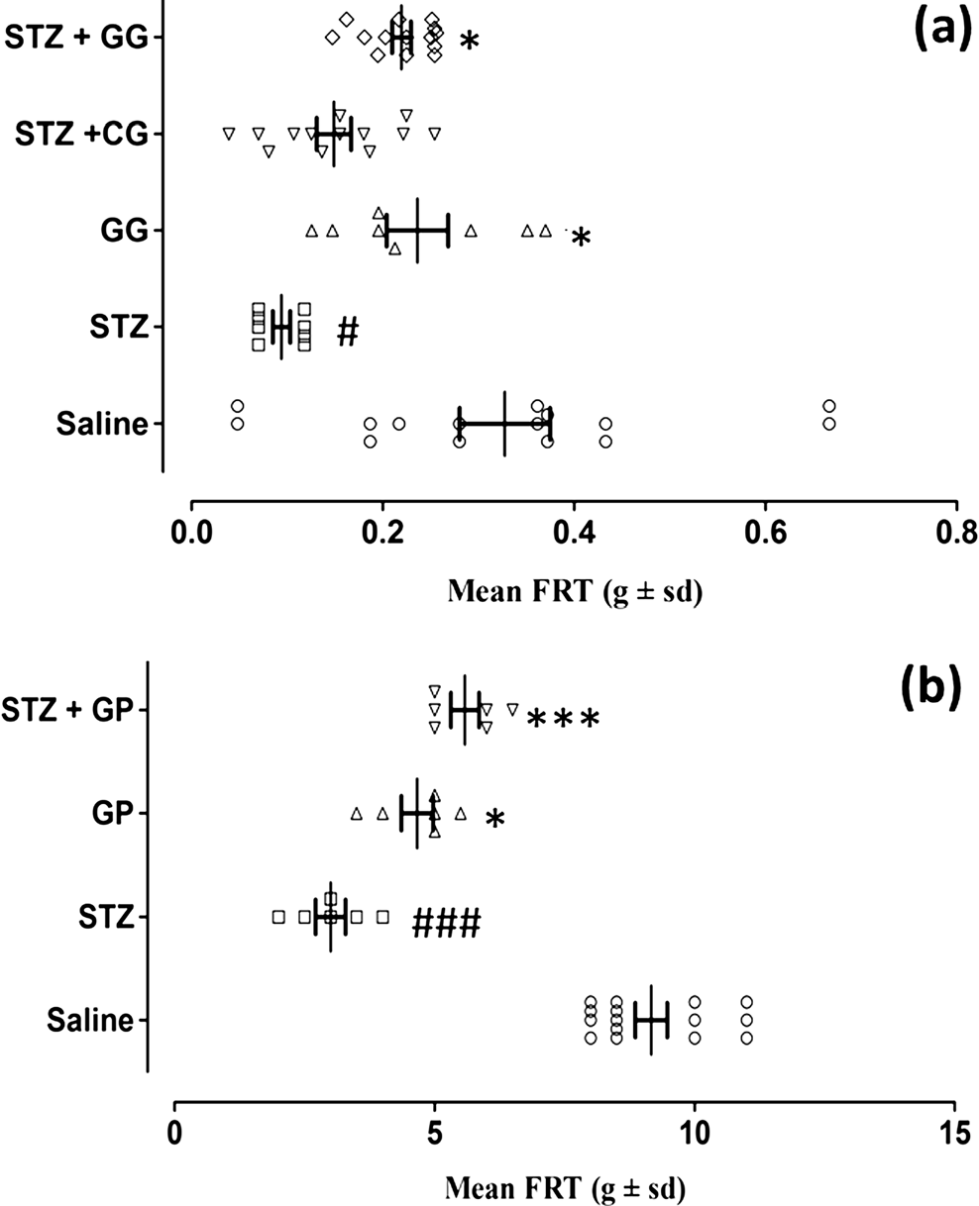
The static antivulvodynia effect of gabapentin gel (10 %) and systemic gabapentin (75 mg/kg i.p.) in streptozotocin-induced diabetic female rats. Scatter plots showing mean von Frey filament force flinching response thresholds (FRTs, *g* ± SD) were determined in all groups (*n* = animal group numbers meeting the inclusion criteria are shown in *brackets*). **a** Topical control gabapentin gel (10 %) alone without streptozotocin pretreatment (*triangle*, GG, *n* = 8) or streptozotocin pretreatment with either control gel (*inverted triangle*, STZ + CG, *n* = 13) or with gabapentin gel (*diamond*, STZ + GG, *n* = 14) was applied on the anogenital area including mons pubis of rats for three times daily and responses were measured 1 h after application. Significance of differences in FRT is shown between topical saline control (*white circle*, *n* = 15) and streptozotocin pretreatment (*white square*, STZ, *n* = 8; #*P* < 0.05) and between streptozotocin pretreatment (*white square*, STZ, *n* = 8) versus gabapentin gel alone (*triangle*, GG, *n* = 8;*P < 0.05), streptozotocin pretreatment followed by control gel (*inverted triangle*, STZ + CG, *n* = 13; *P > 0.05) and streptozotocin pretreatment followed by gabapentin gel (*diamond*, STZ + GG, *n* = 14; *P < 0.05) application, ANOVA followed by Tukey’s post hoc test. **b** In the systemic study, mean von Frey filament force flinching response thresholds (FRTs, *g* ± SD) were determined in all groups, and gabapentin (*triangle*, GP, 75 mg/kg, *n* = 8) was administered i.p. The responses were measured post-treatment. Statistical significance of differences in FRT is shown between saline control (*white circle*, *n* = 15) and streptozotocin pretreatment (*white square*, STZ, *n* = 8; ###*P* < 0.001) and between streptozotocin pretreatment (*white square*, STZ, *n* = 8) and gabapentin alone (*triangle*, GP, *n* = 8; *P < 0.05), and also from streptozotocin combined with gabapentin (STZ + GP, *inverted triangle*, *n* = 6) post-drug treatment (***P < 0.001), ANOVA with Tukey’s post hoc analysis test

The mean von Frey filament force (*g* ± SD) required to induce an FRT (static vulvodynia) was calculated and then plotted for the five treatment groups at the three merged daily application times (*F*_(4,46)_ = 7.920, *P* < 0.001) shown in Fig. 6a. ANOVA coupled with Tukey’s post hoc analysis revealed a significant reduction in the FRT force expressed after streptozotocin pretreatment compared to the saline-treated group (*P* < 0.05). This decrease was reversed in the group treated with the streptozotocin plus topical gabapentin gel combination (*P* < 0.05) but not in those animals pretreated with streptozotocin plus subsequent control gel. The gabapentin-gel-alone group (GG) did display a difference from animals receiving streptozotocin pretreatment (*P* < 0.05) which was not unexpected (Fig. 6a).

In the systemic gabapentin study, mean von Frey filament pressures (*g*) calculated as FRT values (static vulvodynia) were plotted for treatment groups at the pooled 1 and 2-h intervals (*F*_(3,20)_ = 51.02, *P* < 0.001) shown in Fig. 6b. There was a significant reduction of FRT in the group pretreated with streptozotocin (STZ) compared to the saline-administered group (*P* < 0.001). This action was reversed in the group that received streptozotocin pretreatment plus ensuing gabapentin (STZ + GP, *P* < 0.001). In addition, there was a difference in FRT between the group administered gabapentin alone (GP) versus STZ pretreatment (*P* < 0.05) and this is presented in Fig. 6b.

### Activity of topical gabapentin gel (10 %) and systemic gabapentin (75 mg/kg i.p) on dynamic vulvodynia in female diabetic rats

**Fig. 7:**
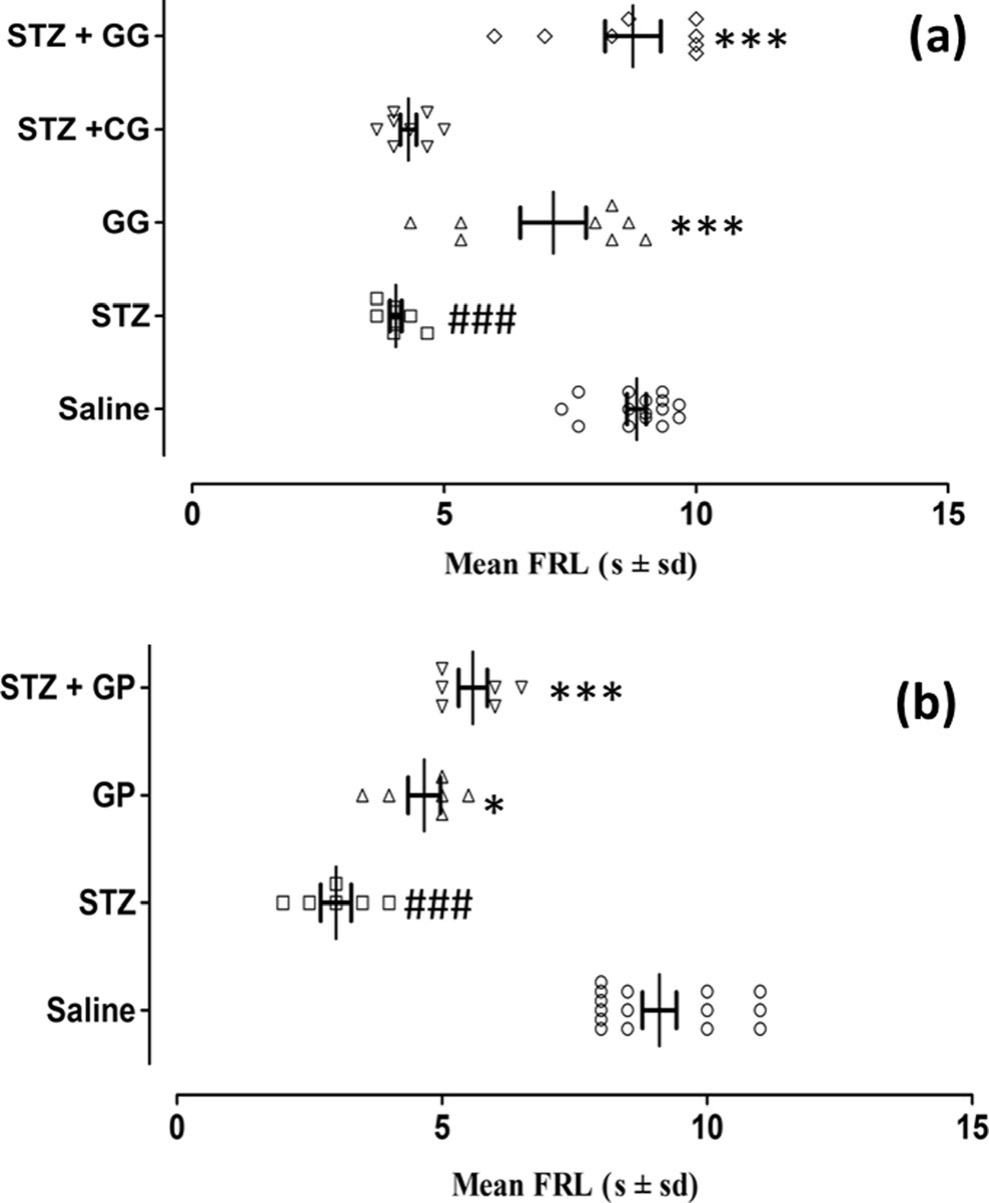
The dynamic antivulvodynia effect of gabapentin gel 10 % and systemic gabapentin (75 mg/kg i.p.) in streptozotocin-induced female diabetic rats. Scatter plots showing mean flinching response latency (FRL, s ± SD) from the anogenital area (vulva) in response to light brushing determined in all groups (*n* = animal group numbers meeting the inclusion criteria are shown in *brackets*). **a** Topical control gabapentin gel alone (*triangle*, GG, *n* = 8), streptozotocin pretreatment followed by gabapentin gel (10 %) (*diamond*, STZ + GG, *n* = 8) or streptozotocin pretreatment followed by control gel (*inverted triangle*, STZ + CG, *n* = 8) was applied to the anogenital area (vulva) of rats for three times daily and FRL was measured 1 h after application. Significance of differences in FRL is shown between topical saline control (*white circle*, *n* = 15) versus streptozotocin pretreatment (*white square*, STZ, *n* = 8; ^###^ *P* < 0.001), gabapentin gel alone versus streptozotocin pretreatment (GG, *triangle*, *n* = 8; ***P < 0.001) and streptozotocin pretreatment followed by gabapentin gel (STZ + GG, *diamond*, *n* = 8, ***P < 0.001), ANOVA with post hoc Tukey’s test. **b** In the systemic study, the flinching response latency (FRL) was determined in each group using the same protocol as for gabapentin gel and this was plotted against treatment groups. The FRL was measured for gabapentin (*triangle*, GP, 75 mg/kg i.p.). ANOVA with Tukey’s post hoc analysis revealed significant differences in FRL between either the control gabapentin-alone group (*triangle*, GP, 75 mg/kg, *n* = 8; *P < 0.05) or the saline controls (*white circle*, *n* = 15) and streptozotocin controls (*white square*, STZ, *n* = 8; ###*P* < 0.001). There were subsequent increases in FRL caused by gabapentin following streptozotocin pretreatment (*inverted triangle*, STZ + GP, *n* = 6; ***P < 0.001)

As depicted in Fig. 7a, the FRL in seconds (dynamic vulvodynia) from the rat anogenital area (vulva) to light brushing was determined in the morning, noon and afternoon pooled groups and then graphed (*F*_(4,35)_ = 31.14, *P* < 0.001). The latency values following saline control treatment were reduced in the streptozotocin-pretreated group (STZ) (*P* < 0.001) and subsequently reversed by combined topical gabapentin gel application (STZ + GG, *P* < 0.001). In the group treated with control gel along with streptozotocin pretreatment (STZ + CG), there was no difference noted in comparison with streptozotocin-alone pretreatment (STZ). Moreover, topical gabapentin gel by itself (GG) produced virtually no change in FRL compared to the saline controls, the latency values additionally being significantly greater than in animals pretreated with streptozotocin (*P* < 0.001, Fig. 7a).

In the systemic study, the FRL values (dynamic vulvodynia) were plotted for merged groups at 1 and 2-h treatment intervals (*F*_(3,20)_ = 51.02, *P* < 0.001). The outcome revealed a significant difference between the latency following saline control treatment which was reduced by streptozotocin pretreatment (STZ) (*P* < 0.001) and subsequently reversed by combined systemic gabapentin treatment (STZ + GP, 75 mg/kg, *P* < 0.001) as shown in Fig. 7b. In addition, the gabapentin-alone treatment group (GP, 75 mg/kg) displayed greater FRL values (*P* < 0.05) than the STZ-pretreated controls in the protocol (Fig. 7b).

## Discussion

Diabetic neuropathy is a common and serious complication stemming from metabolic abnormalities (Elliott 1994) causing lesions of the peripheral and/or central nervous system leading to sensory signs and symptoms (Backonja 2003). It is characterized by progressive chronic neuropathic pain that is tingling and burning in nature with hyperesthesia and paresthesia with deep aching and it is increased by touch (Krause and Backonja 2003). It is greater at night (Boulton et al. 2005) and occurs in the feet and lower legs and may involve the hands (Vinik et al. 2000) affecting patient sleep, mood, self-esteem, social life (Said 2007) and ability to work (Vileikyte et al. 2003). Neuropathic pain is often associated with allodynia, which is a distinct feature and represents an abnormal pain to a stimulus that does not normally provoke pain (Merskey 1986). It has also been reported that small unmyelinated and large myelinated primary nerves (Aβ and small-diameter nociceptive fibres) are implicated in allodynia (Field et al. 1999). Vulvodynia has features which are characteristic of other chronic neuropathic pain conditions. These include the persistent and burning quality of the pain, the allodynia and hyperpathia, the absence of physical findings other than erythema on examination, and the patient’s obsession with the pain (Ben-David and Friedman 1999).

Experimental diabetes mellitus can be induced by most commonly employed diabetogenic agents, i.e. alloxan and streptozotocin. However, streptozotocin-induced diabetes mellitus is used as a model for hyperalgesia (Bishnoi et al. 2011). Therefore, in this study, a diabetic neuropathic pain model of allodynia and vulvodynia was employed involving a single injection of streptozotocin (50 mg/kg) in female rats. Since streptozotocin exhibits a stability problem (Rakieten et al. 1963), fresh solutions were prepared at the time of administration, and diabetes was confirmed by resultant degenerative changes in the pancreatic islets of Langerhans (Fig. 1).

Early neuropathic symptoms in streptozotocin-induced diabetic rodents have been reported including allodynia and decreased nerve function velocity as well as axonal dystrophy following electron microscopical analysis (Cameron et al. 1994, 1999; Li et al. 2004; Weiss et al. 1995). Moreover, later symptoms of streptozotocin neuropathy, some of which are similarly insulin reversible, have been well documented and consist of hypoalgesia, large sensory nerve fibre pathology comprising demyelination, degeneration and Schwann cell damage and regeneration (Calcutt et al. 2004; Muller et al. 2008; Shaikh and Somani 2011; Vasconcelos et al. 2011; Weiss et al. 1995). Furthermore, in streptozotocin-treated rats, bladder afferent pudendal neurons located in the L6 and S1 dorsal root ganglia (McKenna and Nadelhaft 1986) have been reported to be smaller in cross-sectional area in streptozotocin-induced diabetics than in normal animals (Steers et al. 1994) indicating axonopathy (Chen et al. 2013).

The current management approach to vulvodynia is redolent of neuropathic pain treatment, usually involving adjunctive therapy since monotherapy often fails to yield an effective response. Pharmacological and non-pharmacological interventions include oral and topical medications, local and regional injections, cognitive therapy, physical therapy and surgery (Andrews 2010; Bates and Timmins 2002). Literature reports regarding evidence of efficacy for pain reduction by pharmacological intervention of generalized unprovoked vulvodynia vary from fair to poor. The majority of these reports lack high-quality evidence (Andrews 2010). However, adequate substantiation of efficacy has been reported for xylocaine (5 %), oral pregabalin, oral or topical gabapentin, oral duloxetine and selective serotonin reuptake inhibitors (SSRIs). Moreover, there is evidence of poor efficacy for topical capsaicin, nitroglycerin, oral tricyclic antidepressants or venlafaxine, pentosan polysulfate, opioids, tramadol, carbamazepine, lamotrigine, oxcarbazepine, topiramate and valproic acid (Andrews 2010; Fischer 2004; Phillips and Bachmann 2010). Based on these promising case reports and retrospective investigations previously performed on allodynia and vulvodynia, it was decided to conduct systemic and topical gabapentin studies in order to validate streptozotocin-induced diabetes as an animal model of vulvodynia.

Arising from a pilot study, the streptozotocin-administered animals were kept under strict observation for 29 days to develop robust symptoms of static and dynamic allodynia and vulvodynia in the animal model. The inclusion criteria for animals in each group are depicted in Table 1. Static allodynia and vulvodynia were present in some of the animals within 15 days following streptozotocin injection, while both dynamic allodynia and vulvodynia were detected in most subjects within 29 days post-streptozotocin injection. Overall assessment at different times indicated that there were exclusions of animals at different stages up to protocol day 29 in order to achieve the criteria for the paradigm. The streptozotocin-treated animals also showed significant loss in body weight (Fig. 3) with elevated blood glucose levels (i.e. above 270 mg/dL) as compared to saline-vehicle-treated animals (Fig. 2). In order to test for allodynia and vulvodynia, in the topical gel study, the animals were divided into treatment groups: saline control, streptozotocin pretreatment control, control gabapentin gel alone without streptozotocin pretreatment, streptozotocin pretreatment plus control gel vehicle and streptozotocin pretreatment plus gabapentin gel (10 %). In the case of systemic gabapentin (75 mg/kg) for allodynia and vulvodynia, the animals were divided into saline control, streptozotocin pretreatment control, gabapentin alone without streptozotocin pretreatment and streptozotocin pretreatment plus gabapentin treatment groups. Responses were measured by application of von Frey filaments or light brushing with cotton buds. Streptozotocin itself induced explicit static and dynamic mechanical allodynia and vulvodynia in comparison with saline-treated controls. This was evidenced by significantly reduced von Frey filament force PWT, reduced PWL, vulval FRT and FRL. Control and gabapentin gels were applied in the morning, noon and afternoon on the hind paws and anogenital (vulva) area of animals, while gabapentin (75 mg/kg) was administered intraperitoneally (i.p.) for subsequent allodynia and vulvodynia testing, respectively. The results showed that topically applied gabapentin gel was able to ameliorate static and dynamic allodynia in the streptozotocin-pretreated animals (Suppl Figs. 4a and 5a). Systemic gabapentin administration showed similar outcomes by alleviating static and dynamic allodynia as compared to streptozotocin-pretreated controls (Suppl Figs. 4b and 5b). In the case of static and dynamic vulvodynia, there was a statistically significant reversal of streptozotocin-induced vulvodynia of both types by topical gabapentin gel application (Suppl Figs. 6a and 7a). Likewise, systemic gabapentin against static and dynamic vulvodynia displayed significant differences between the combined gabapentin/streptozotocin and streptozotocin-alone group values reflecting symptom reversal (Suppl Figs. 6b and 7b). Vulvodynia is a chronic condition; however, testing in the model was performed on day 29 in the protracted protocol and not on days 5 and 15 for the purpose of avoiding intervening repeated-test stress induction. Consequently, the persistent quality of vulvodynia during this interim period was not fully ascertained. Thus, systemic administration of gabapentin has provided some evidence with respect to validation of the current animal model for allodynia and vulvodynia since this agent has exhibited efficacy not only in animals (Carlton and Zhou 1998; Field et al. 1999) but also in the clinic (Boardman et al. 2008; Gorson et al. 1999).

In the gabapentin topical study, there was an apparent antiallodynia effect of the control gel seen in this investigation. This might derive from the finding that streptozotocin induces epidermal thinning in the plantar surface of the rat hind paw (Kadic et al. 2014), and the occlusive coating quality of the xanthan gum hydrocolloid constituent (Marchitto and Flock 2013) along with the polyacrylamide in the gel may have acted as a physical barrier to mechanical stimuli applied to the weakened paw surface. This is somewhat analogous to the hydrocolloid gel in dressings which give rise to patients experiencing less pain and the need for less analgesia in the management of abrasive wounds (Heffernan and Martin 1994) or following skin shave biopsy (Nemeth et al. 1991). Conversely, the lack of alleviation of static and dynamic vulvodynia by the control gel may be a high-level sensory consequence of the density of ventral and dorsal sensory nerve branches emerging from the pudendal canal, respectively, to the perineum (superficial perineal nerve) and genitalia (terminal branch) (Robert et al. 1998). Furthermore, the inability of gabapentin gel to show any significant antiallodynia activity in comparison to control gel may be attributed to restricted permeation of gabapentin from the gel formulation through the paw skin. However, it is noteworthy that gabapentin delivered from control gel does penetrate human trunk skin (PCCA 2012) although this particular skin area is likely to be much thinner than that of the rat paw.

Gabapentin has been indicated for the treatment of neuropathy though the exact mechanism through which it inhibits neuropathic pain still remains unclear. Evidence indicates that gabapentin operates by increasing the level of gamma aminobutaric acid (GABA) (Kocsis and Honmou 1994), by acting as a non-*N*-methyl-d-aspartate (non-NMDA) receptor antagonist (Chen et al. 2000; Kaneko et al. 2000) and by inhibiting the α2δ1 subunit of voltage-gated calcium channels (Gee et al. 1996; Shimoyama et al. 2000). Thus, gabapentin could possibly reduce neuropathic signs via several potential mechanisms. The efficacy for pain reduction by pharmacological intervention of generalized unprovoked vulvodynia varies from fair to poor (Andrews 2010). In this respect, the topical formulation of gabapentin may be a useful addition to the pharmacological treatment options available for neuropathic pain syndromes. The topical gabapentin 10 % gel preparation tested in this study is likely to possess minimal adverse effects compared to those usually associated with systemic use of gabapentin such as sedation, dizziness, somnolence, asthenia, ataxia, amblyopia, headache and nausea (McLean et al. 2006). This may be ascribed to the lower systemic concentration of active ingredient delivered by the topical formulation than that yielded by oral administration. Therefore, topical use of gabapentin in the form of a gel could be a better alternative for the clinical management of neuropathic pain syndromes such as allodynia and vulvodynia.

## Conclusions

In summary, these findings on the outcomes of topical and systemic gabapentin in the diabetic neuropathy model of vulvodynia tend to validate it as a useful non-clinical paradigm. Nonetheless, the complex nature of the diabetic neuropathic pain syndrome and the unpredictable rate of absorption of gabapentin from the gel formulation warrant further research to correlate any antivulvodynia activity of gabapentin gel with gabapentin penetration after topical application. The antivulvodynia action of gabapentin gel in the current model also accords with retrospective clinical studies which concluded that topical gabapentin is well tolerated and associated with significant pain relief in women with vulvodynia (Andrews 2010; Boardman et al. 2008).

## Electronic supplementary material

Fig. 4 The static anti-allodynia activity of gabapentin gel (10 %) and systematic gabapentin (75 mg /kg i.p.) in streptozotocin-induced female diabetic rats. Scatter plots showing mean paw withdrawal thresholds (PWT, g ± sd) in response to von Frey hairs were determined in all groups. (**a**) Gabapentin gel (10 %) alone without streptozotocin pretreatment (GG, *n* = 8), streptozotocin pretreatment followed by control gel (STZ + CG, *n* = 9) or streptozotocin pretreatment followed by gabapentin gel (STZ + CG, *n* = 5) were applied on plantar surface of the right hind paws of rats three times daily at the times shown and PWT was measured 1 hour later. (Significance of differences in PWT are shown between saline (SAL, *n* = 15) vs streptozotocin control (STZ, *n* = 6; ^***^ *P* < 0.001) and between streptozotocin control (STZ, *n* = 6) vs gabapentin gel alone without streptozotocin pretreatment (GG, *n* = 8; ^**^ *P* < 0.01), streptozotocin pretreatment with control gel (STZ + CG, *n* = 9; ^**^ *P* < 0.01, ^***^ *P* < 0.001) and streptozotocin pretreatment plus gabapentin gel (STZ + GG, *N* = 5; ^***^ *P* < 0.001), ANOVA followed by Tukey’s *post hoc* test). (**b**) In the case of the systematic study, gabapentin (75 mg/kg) was administered intraperitoneally (i.p.) and PWT was measured using the same protocol as gabapentin gel at 1 and 2 hours post treatment. (ANOVA followed by Tukey’s *post hoc* test revealed statistical differences in PWT between saline (SAL, *n* = 15) vs streptozotocin control (STZ, *n* = 6; ^**^ *P* < 0.01, ^***^ *P* < 0.001), between streptozotocin control (STZ, *n* = 6) vs gabapentin alone without streptozotocin pretreatment (GP 75 mg/kg, *n* = 5; ^*^ *P* < 0.05. ^**^ *P* < 0.01) and also between streptozotocin plus gabapentin (STZ + GP 75 mg/kg, *n* = 8; ^*^ *P* < 0.05). (GIF 154 kb)

High resolution image (TIFF 97 kb) High resolution image (TIFF 57 kb)

Fig. 5 The dynamic anti-allodynia activity of gabapentin gel 10 % and systemic gabapentin (75 mg /kg i.p.) in streptozotocin-induced female diabetic rats. Scatter plots showing mean paw withdrawal latencies (PWL, s ± sd) in response to light brushing were determined in all groups (*n* = animal group numbers meeting the inclusion criteria are shown in brackets). (**a**) Control gabapentin gel alone without streptozotocin treatment (GG, *n* = 8), streptozotocin treatment followed by control gel (STZ + CG, *n* = 7) were applied on plantar surface of the right hind paw of rats three times daily at the times shown and PWL was measured 1 hour later. (Significance of differences in PWL are shown between saline (SAL, *n* = 15) vs streptozotocin control (STZ, *n* = 6; ^***^ *P* < 0.001) and from streptozotocin control (STZ, *n* = 6) versus gabapentin gel alone without STZ pretreatment (*n* = 8; ^**^ *P* < 0.001. ^***^ *P* < 0.001), streptozotocin treatment (*n* = 8; ^**^ *P* < 0.01, ^***^ *P* < 0.001), streptozotocin treatment followed by control gel i.e STZ + CG (*n* = 7; ^**^ *P* < 0.01, ^***^ *P* < 0.001) and streptozotocin treatment followed by gabapentin gel i.e STZ + GG (10 %) (*n* = 10; ^***^ *P* < 0.001), ANOVA followed by Tukey's *post hoc* test). (**b**) In the case of the systemic study, the same protocol as topical gabapentin was used but gabapentin (GP, 75 mg/kg) was administered i.p and PWL was measured at 1 and 2 hours post treatment. (Statistical significance of differences in PWL are shown between saline (SAL, *n* = 15) vs streptozotocin control (STZ, *n* = 6) (^**^ *P* < 0.01, ^***^ *P* < 0.001) and from streptozotocin control (STZ, *n* = 6) versus gabapentin alone without STZ pretreatment (GP, *n* = 8; ^*^ *P* < 0.05) and STZ + GP 75 mg/kg (*n* = 5; ^*^ *P* < 0.05, ^**^ *P* < 0.01). (GIF 236 kb) (GIF 157 kb)

High resolution image (TIFF 94 kb) High resolution image (TIFF 57 kb)

Fig. 6 The static anti-vulvodynia sffect of gabapentin gel (10 %) and systemic gabapentin (75 mg /kg i.p.) in streptozotocin-induced diabetic female rats. Scatter plots showing mean von Frey hair force flinching response thresholds (FRT, g ± sd) were determined in all groups (*n* = animal group numbers meeting the inclusion criteria are shown in brackets). (**a**) Control gabapentin gel alone without streptozotocin pretreatment (GG, *n* = 8), streptozotocin pretreatment with either control gel (STZ + CG, *n* = 13) or gabapentin gel (10 %) (STZ + GG, *n* = 14) were applied on the anogenital area including mons pubis of rats three times daily at the times shown and responses were measured 1 hour later. (Significance of differences in FRT between saline control (SAL, *n* = 15) vs streptozotocin control (STZ, *n* = 8; ^**^ *P* < 0.01, ^***^ *P* < 0.001) and between streptozotocin control (STZ, *n* = 8) vs gabapentin gel alone without streptozotocin pretreatment (GG, *n* = 8; ^*^ *P* < 0.05, ^**^ *P* < 0.01), streptozotocin pretreatment followed by control gel i.e. STZ + CG (*n* = 13; *P* > 0.05) and streptozotocin pretreatment followed by gabapentin gel i.e. STZ + GG (*n* = 14; ^*^ *P* < 0.05) application, ANOVA followed by Tukey’s *post hoc* test). (**b**) In the systemic study, mean von Frey hair force flinching response thresholds (FRT, g ± sd) were determined in all groups and gabapentin (GP, 75 mg/kg, *n* = 8) was administered i.p. The responses were measured post treatment at 1 and 2 hours. (Statistical significance of differences in FRT were between saline control (SAL, *n* = 15) vs streptozotocin control (STZ, *n* = 8; ^**^ *P* < 0.01) and from streptozotocin control (STZ, *n* = 8) vs gabapentin alone without streptozotocin pretreatment (GP, *n* = 8; ^*^ *P* < 0.05, ^**^ *P* < 0.01), STZ + GP (*n* = 6) at 1 and 2 hours post drug treatment (^*^ *P* < 0.05), ANOVA with Tukey’s *post hoc* analysis test). (GIF 204 kb) (GIF 154 kb)

High resolution image (TIFF 92 kb) High resolution image (TIFF 55 kb)

Fig. 7 The dynamic anti-vulvodynia effect of gabapentin gel 10 % and systemic gabapentin (75 mg /kg i.p.) in streptozotocin-induced female diabetic rats. Scatter plots showing mean flinching response latencies (FRL, s ± sd) of the anogenital area (vulva) in response to light brushing were determined in all groups (*n* = animal group numbers meeting the inclucion criteria are shown in brackets). (**a**) Control gabapentin gel alone without streptozotocin pretreatment (GG, *n* = 8), streptozotocin pretreatment followed by gabapentin gel (10 %) (STZ + GG, *n* = 8) or streptozotocin pretreatment followed by contron gel (STZ + CG, *n* = 8) were applied to the anogenital area (vulva) of rats three time daily at the times shown and FRL was measured 1 hour later. (Significance of differences in FRL were saline control (SAL, *n* = 15) vs streptozotocin control (STZ, *n* = 8; ^***^ *P* < 0.001), gabapentin gel without streptozotocin pretreatment (*n* = 8) vs streptozotocin control STZ (*n* = 8; ^*^ *P* < 0.05, ^**^ *P* < 0.01) and streptozotocin pretreatment followed by gabapentin gel (STZ + GG, *n* = 8) treatments in the morning (^**^ *P* < 0.01), noon and afternoon (^***^ *P* < 0.001), ANOVA with *post hoc* Tukey’s test). (**b**) In the systemic study, the flinching response latencies (FRL) were determined in each group using the same protocol as for gabapentin gel and was plotted against post administration time (hours). The FRL was measured at 1 and 2 hours post gabapentin (GP, 75 mg /kg i.p.). ANOVA with Tukey’s *post hoc* analysis revealed significances in FRL between either the control gabapentin alone without streptozotocin pretreatment group (GP, 75 mg/kg, *n* = 8; ^*^ *P* < 0.05, ^**^ *P* < 0.01) or saline controls (SAL, *n* = 15) vs streptozotocin controls (STZ, *n* = 8; ^**^ *P* < 0.01). There were subsequent increases in FRL caused by gabapentin following streptozotocin pretreatment (STZ + GP, *n* = 6) at 1 hour (^*^ *P* < 0.05) and 2 hours (^*^ *P* < 0.05). (GIF 206 kb) (GIF 170 kb)

High resolution image (TIFF 92 kb) High resolution image (TIFF 62 kb)
