# Supplementary figures and images for: A streptozotocin-induced diabetic neuropathic pain model for static or dynamic mechanical allodynia and vulvodynia: validation using topical and systemic gabapentin
Source: Naunyn Schmiedebergs Arch Pharmacol. 2015 Jul 3;388(11):1129–40. doi: 10.1007/s00210-015-1145-y (PMC4619463; doi:10.1007/s00210-015-1145-y)

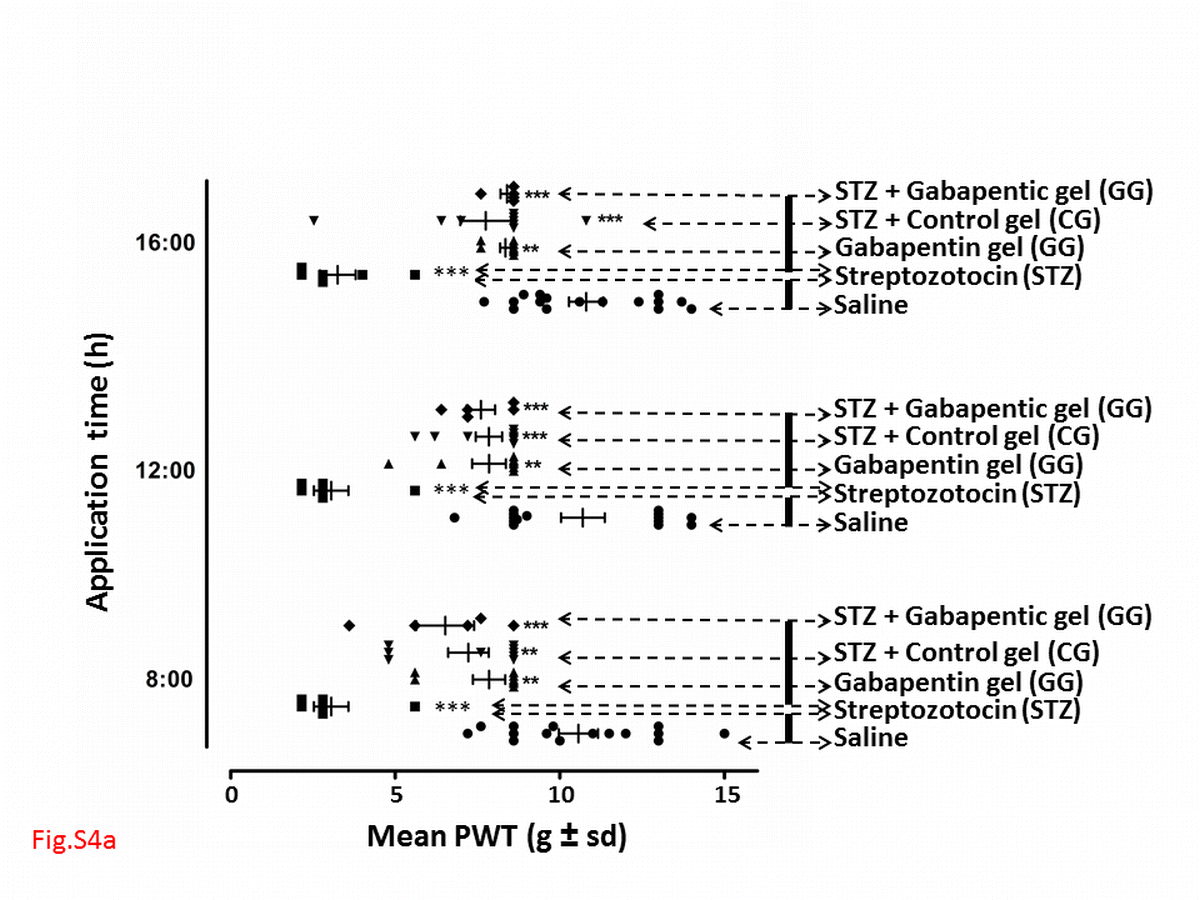

Supplement: Fig. 4 — The static anti-allodynia activity of gabapentin gel (10 %) and systematic gabapentin (75 mg /kg i.p.) in streptozotocin-induced female diabetic rats. Scatter plots showing mean paw withdrawal thresholds (PWT, g ± sd) in response to von Frey hairs were determined in all groups. (a) Gabapentin gel (10 %) alone without streptozotocin pretreatment (GG, n = 8), streptozotocin pretreatment followed by control gel (STZ + CG, n = 9) or streptozotocin pretreatment followed by gabapentin gel (STZ + CG, n = 5) were applied on plantar surface of the right hind paws of rats three times daily at the times shown and PWT was measured 1 hour later. (Significance of differences in PWT are shown between saline (SAL, n = 15) vs streptozotocin control (STZ, n = 6; *** P < 0.001) and between streptozotocin control (STZ, n = 6) vs gabapentin gel alone without streptozotocin pretreatment (GG, n = 8; ** P < 0.01), streptozotocin pretreatment with control gel (STZ + CG, n = 9; ** P < 0.01, *** P < 0.001) and streptozotocin pretreatment plus gabapentin gel (STZ + GG, N = 5; *** P < 0.001), ANOVA followed by Tukey’s post hoc test). (b) In the case of the systematic study, gabapentin (75 mg/kg) was administered intraperitoneally (i.p.) and PWT was measured using the same protocol as gabapentin gel at 1 and 2 hours post treatment. (ANOVA followed by Tukey’s post hoc test revealed statistical differences in PWT between saline (SAL, n = 15) vs streptozotocin control (STZ, n = 6; ** P < 0.01, *** P < 0.001), between streptozotocin control (STZ, n = 6) vs gabapentin alone without streptozotocin pretreatment (GP 75 mg/kg, n = 5; * P < 0.05. ** P < 0.01) and also between streptozotocin plus gabapentin (STZ + GP 75 mg/kg, n = 8; * P < 0.05). [file 210_2015_1145_Fig8_ESM.gif]

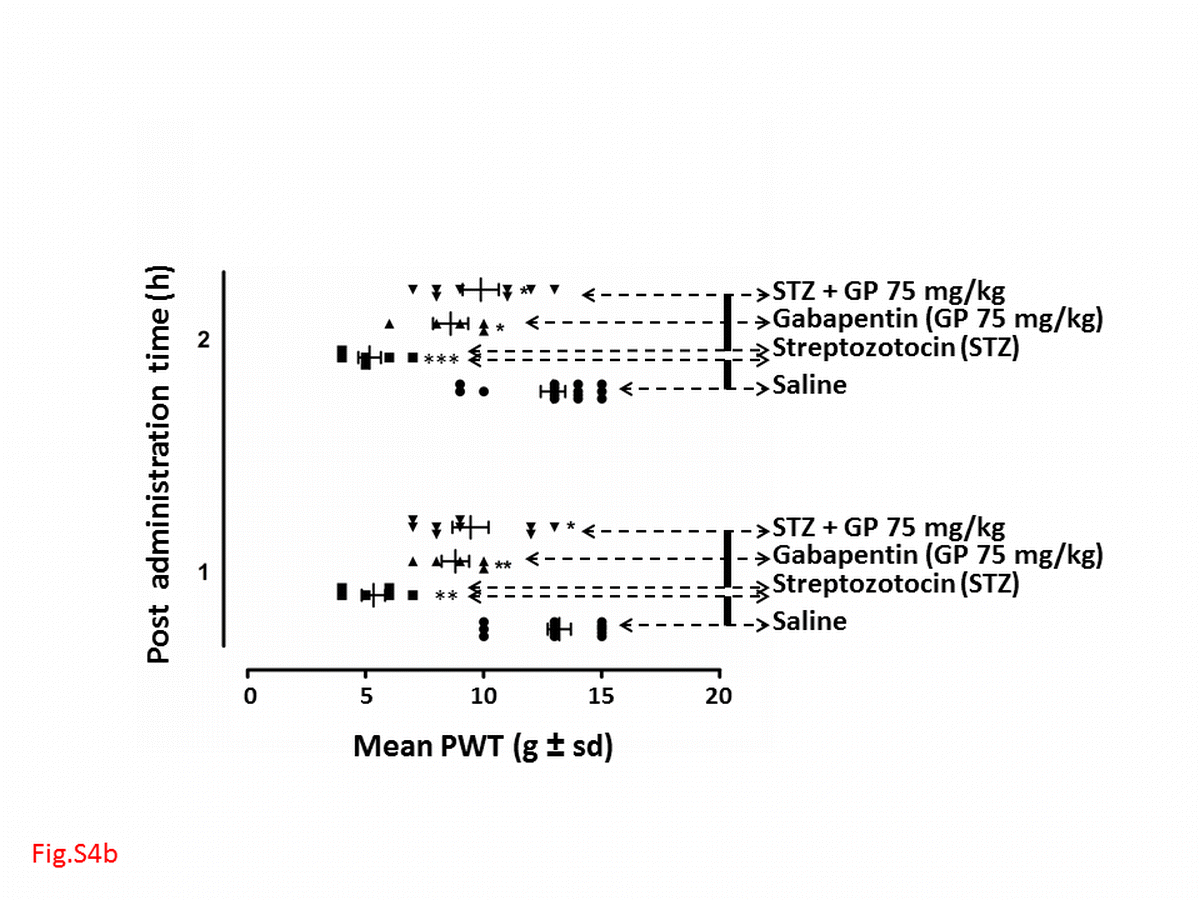

Supplement: Fig. 4 — The static anti-allodynia activity of gabapentin gel (10 %) and systematic gabapentin (75 mg /kg i.p.) in streptozotocin-induced female diabetic rats. Scatter plots showing mean paw withdrawal thresholds (PWT, g ± sd) in response to von Frey hairs were determined in all groups. (a) Gabapentin gel (10 %) alone without streptozotocin pretreatment (GG, n = 8), streptozotocin pretreatment followed by control gel (STZ + CG, n = 9) or streptozotocin pretreatment followed by gabapentin gel (STZ + CG, n = 5) were applied on plantar surface of the right hind paws of rats three times daily at the times shown and PWT was measured 1 hour later. (Significance of differences in PWT are shown between saline (SAL, n = 15) vs streptozotocin control (STZ, n = 6; *** P < 0.001) and between streptozotocin control (STZ, n = 6) vs gabapentin gel alone without streptozotocin pretreatment (GG, n = 8; ** P < 0.01), streptozotocin pretreatment with control gel (STZ + CG, n = 9; ** P < 0.01, *** P < 0.001) and streptozotocin pretreatment plus gabapentin gel (STZ + GG, N = 5; *** P < 0.001), ANOVA followed by Tukey’s post hoc test). (b) In the case of the systematic study, gabapentin (75 mg/kg) was administered intraperitoneally (i.p.) and PWT was measured using the same protocol as gabapentin gel at 1 and 2 hours post treatment. (ANOVA followed by Tukey’s post hoc test revealed statistical differences in PWT between saline (SAL, n = 15) vs streptozotocin control (STZ, n = 6; ** P < 0.01, *** P < 0.001), between streptozotocin control (STZ, n = 6) vs gabapentin alone without streptozotocin pretreatment (GP 75 mg/kg, n = 5; * P < 0.05. ** P < 0.01) and also between streptozotocin plus gabapentin (STZ + GP 75 mg/kg, n = 8; * P < 0.05). [file 210_2015_1145_Fig9_ESM.gif]

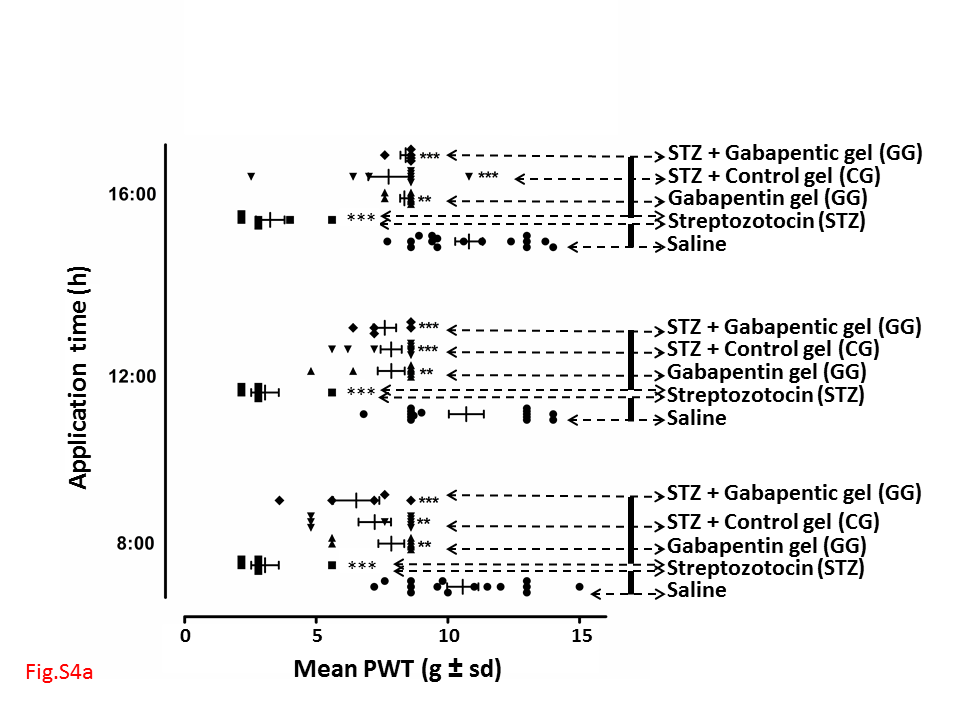

Supplement: Supplementary file 3 — High resolution image (TIFF 97 kb) [file 210_2015_1145_MOESM1_ESM.tif]

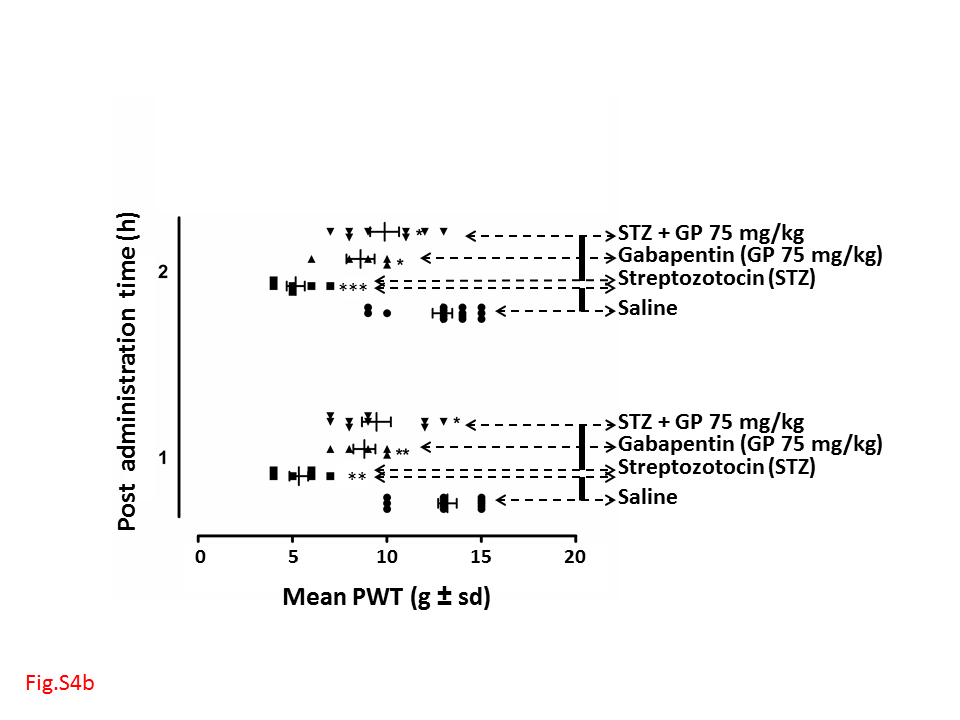

Supplement: Supplementary file 4 — High resolution image (TIFF 57 kb) [file 210_2015_1145_MOESM2_ESM.tif]

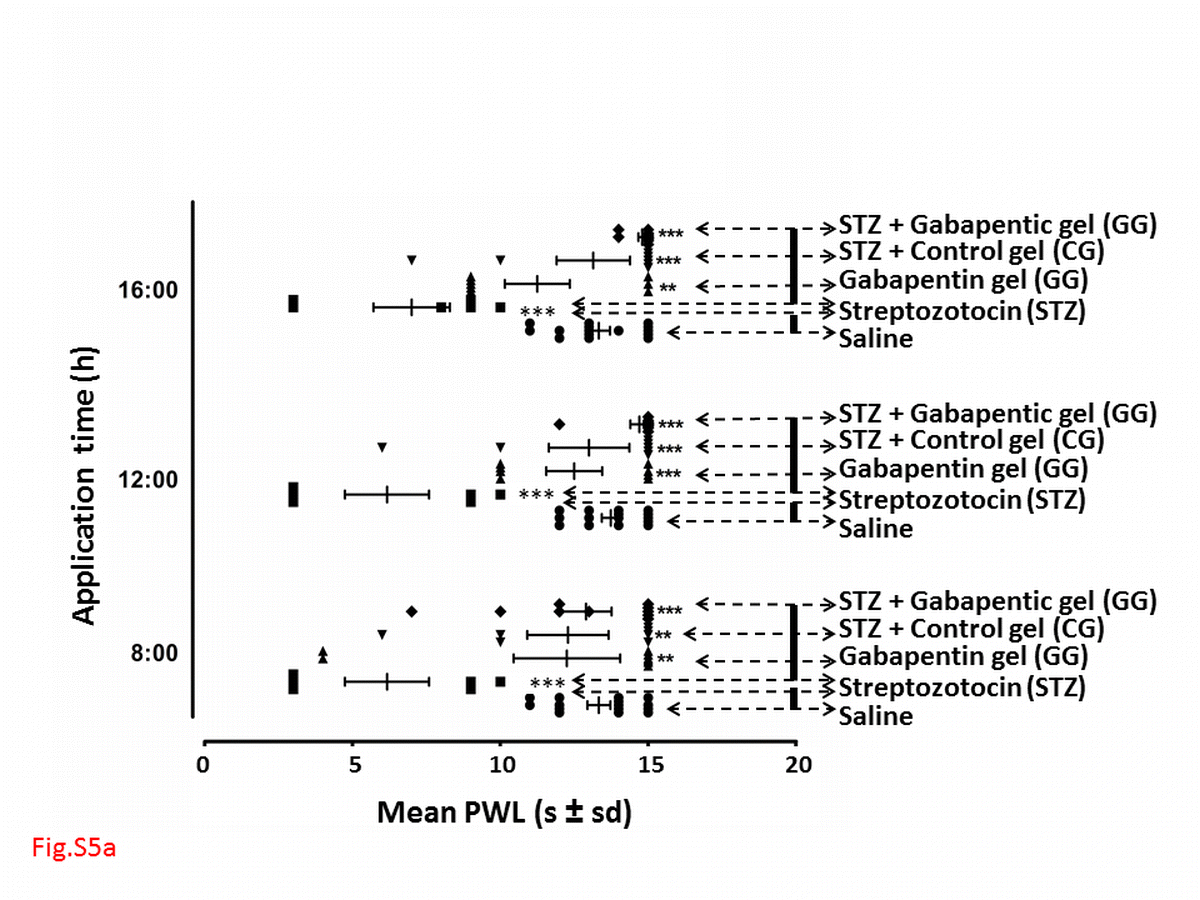

Supplement: Fig. 5 — The dynamic anti-allodynia activity of gabapentin gel 10 % and systemic gabapentin (75 mg /kg i.p.) in streptozotocin-induced female diabetic rats. Scatter plots showing mean paw withdrawal latencies (PWL, s ± sd) in response to light brushing were determined in all groups (n = animal group numbers meeting the inclusion criteria are shown in brackets). (a) Control gabapentin gel alone without streptozotocin treatment (GG, n = 8), streptozotocin treatment followed by control gel (STZ + CG, n = 7) were applied on plantar surface of the right hind paw of rats three times daily at the times shown and PWL was measured 1 hour later. (Significance of differences in PWL are shown between saline (SAL, n = 15) vs streptozotocin control (STZ, n = 6; *** P < 0.001) and from streptozotocin control (STZ, n = 6) versus gabapentin gel alone without STZ pretreatment (n = 8; ** P < 0.001. *** P < 0.001), streptozotocin treatment (n = 8; ** P < 0.01, *** P < 0.001), streptozotocin treatment followed by control gel i.e STZ + CG (n = 7; ** P < 0.01, *** P < 0.001) and streptozotocin treatment followed by gabapentin gel i.e STZ + GG (10 %) (n = 10; *** P < 0.001), ANOVA followed by Tukey's post hoc test). (b) In the case of the systemic study, the same protocol as topical gabapentin was used but gabapentin (GP, 75 mg/kg) was administered i.p and PWL was measured at 1 and 2 hours post treatment. (Statistical significance of differences in PWL are shown between saline (SAL, n = 15) vs streptozotocin control (STZ, n = 6) (** P < 0.01, *** P < 0.001) and from streptozotocin control (STZ, n = 6) versus gabapentin alone without STZ pretreatment (GP, n = 8; * P < 0.05) and STZ + GP 75 mg/kg (n = 5; * P < 0.05, ** P < 0.01). (GIF 236 kb) [file 210_2015_1145_Fig10_ESM.gif]

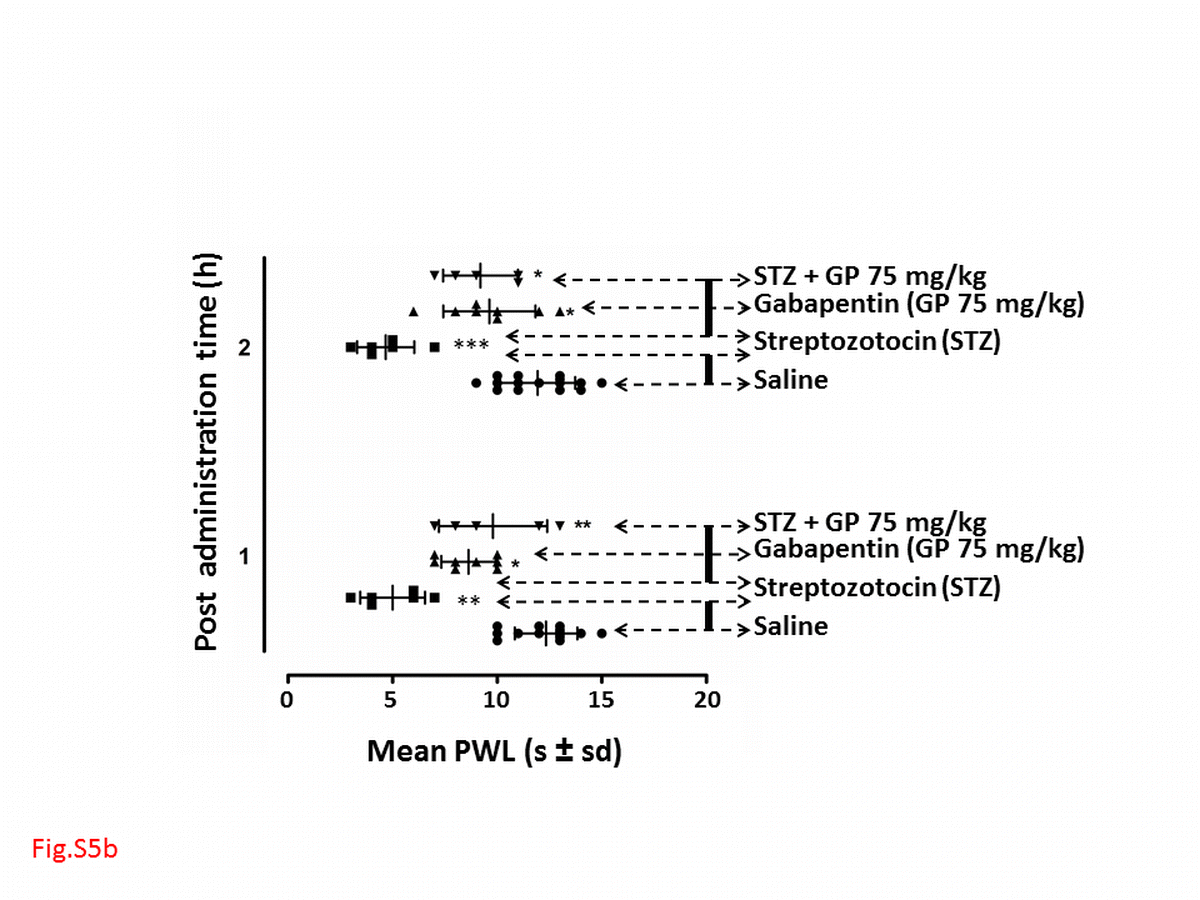

Supplement: Fig. 5 — The dynamic anti-allodynia activity of gabapentin gel 10 % and systemic gabapentin (75 mg /kg i.p.) in streptozotocin-induced female diabetic rats. Scatter plots showing mean paw withdrawal latencies (PWL, s ± sd) in response to light brushing were determined in all groups (n = animal group numbers meeting the inclusion criteria are shown in brackets). (a) Control gabapentin gel alone without streptozotocin treatment (GG, n = 8), streptozotocin treatment followed by control gel (STZ + CG, n = 7) were applied on plantar surface of the right hind paw of rats three times daily at the times shown and PWL was measured 1 hour later. (Significance of differences in PWL are shown between saline (SAL, n = 15) vs streptozotocin control (STZ, n = 6; *** P < 0.001) and from streptozotocin control (STZ, n = 6) versus gabapentin gel alone without STZ pretreatment (n = 8; ** P < 0.001. *** P < 0.001), streptozotocin treatment (n = 8; ** P < 0.01, *** P < 0.001), streptozotocin treatment followed by control gel i.e STZ + CG (n = 7; ** P < 0.01, *** P < 0.001) and streptozotocin treatment followed by gabapentin gel i.e STZ + GG (10 %) (n = 10; *** P < 0.001), ANOVA followed by Tukey's post hoc test). (b) In the case of the systemic study, the same protocol as topical gabapentin was used but gabapentin (GP, 75 mg/kg) was administered i.p and PWL was measured at 1 and 2 hours post treatment. (Statistical significance of differences in PWL are shown between saline (SAL, n = 15) vs streptozotocin control (STZ, n = 6) (** P < 0.01, *** P < 0.001) and from streptozotocin control (STZ, n = 6) versus gabapentin alone without STZ pretreatment (GP, n = 8; * P < 0.05) and STZ + GP 75 mg/kg (n = 5; * P < 0.05, ** P < 0.01). (GIF 236 kb) [file 210_2015_1145_Fig11_ESM.gif]

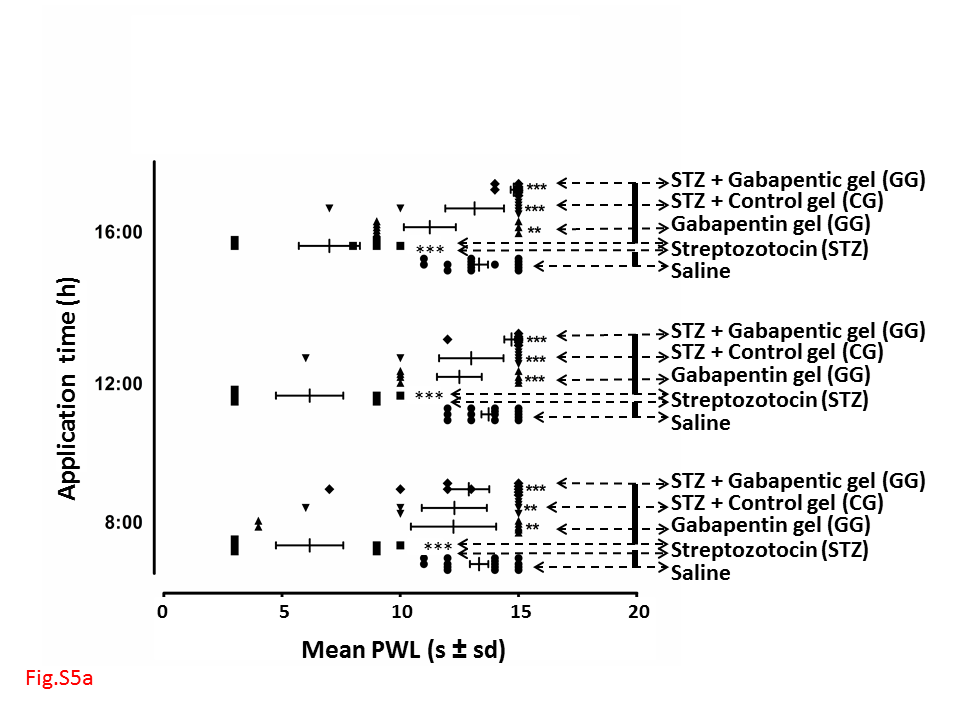

Supplement: Supplementary file 7 — High resolution image (TIFF 94 kb) [file 210_2015_1145_MOESM3_ESM.tif]

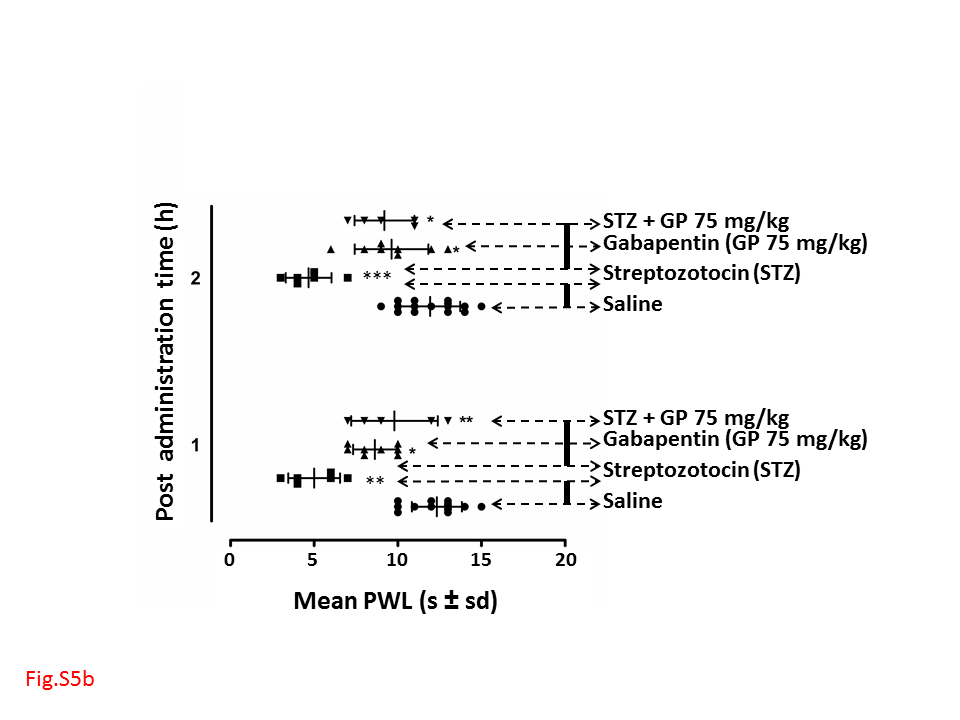

Supplement: Supplementary file 8 — High resolution image (TIFF 57 kb) [file 210_2015_1145_MOESM4_ESM.tif]

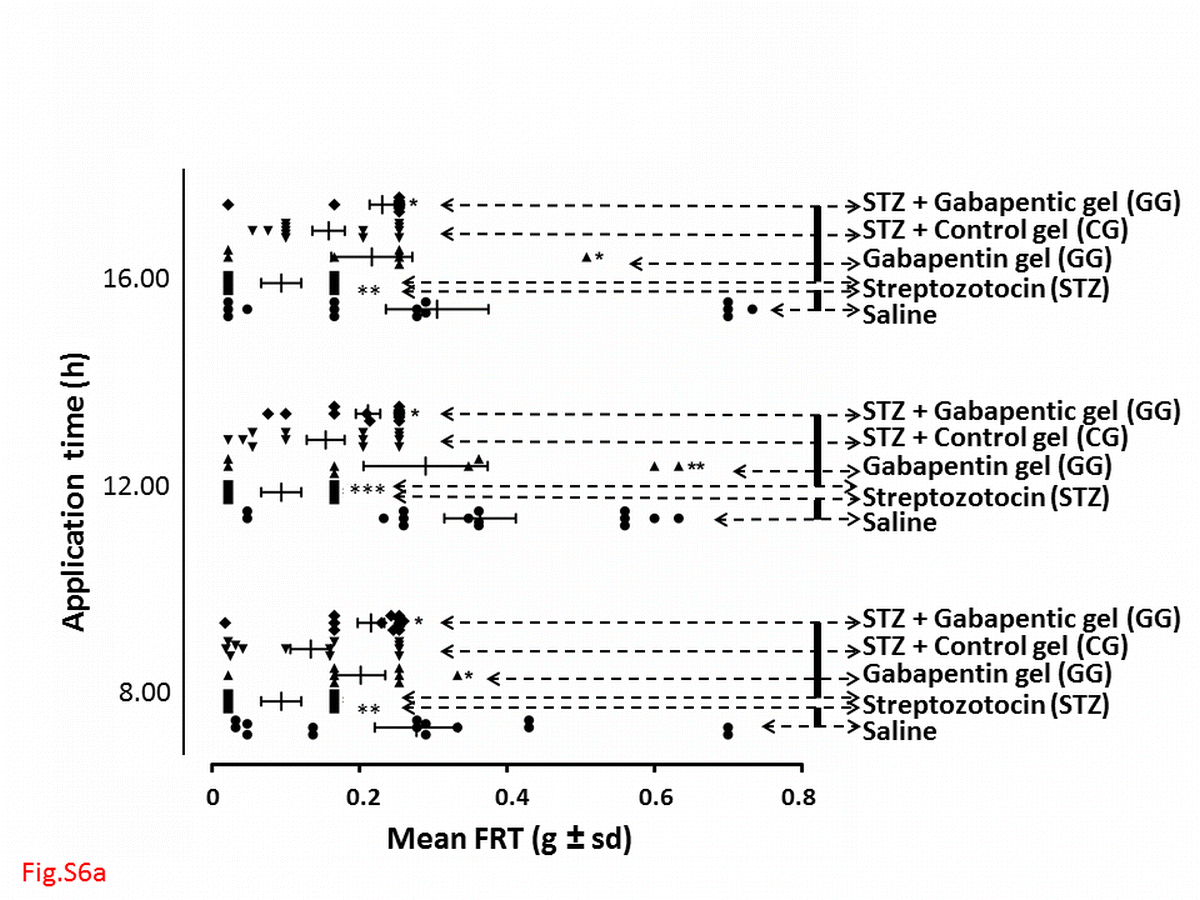

Supplement: Fig. 6 — The static anti-vulvodynia sffect of gabapentin gel (10 %) and systemic gabapentin (75 mg /kg i.p.) in streptozotocin-induced diabetic female rats. Scatter plots showing mean von Frey hair force flinching response thresholds (FRT, g ± sd) were determined in all groups (n = animal group numbers meeting the inclusion criteria are shown in brackets). (a) Control gabapentin gel alone without streptozotocin pretreatment (GG, n = 8), streptozotocin pretreatment with either control gel (STZ + CG, n = 13) or gabapentin gel (10 %) (STZ + GG, n = 14) were applied on the anogenital area including mons pubis of rats three times daily at the times shown and responses were measured 1 hour later. (Significance of differences in FRT between saline control (SAL, n = 15) vs streptozotocin control (STZ, n = 8; ** P < 0.01, *** P < 0.001) and between streptozotocin control (STZ, n = 8) vs gabapentin gel alone without streptozotocin pretreatment (GG, n = 8; * P < 0.05, ** P < 0.01), streptozotocin pretreatment followed by control gel i.e. STZ + CG (n = 13; P > 0.05) and streptozotocin pretreatment followed by gabapentin gel i.e. STZ + GG (n = 14; * P < 0.05) application, ANOVA followed by Tukey’s post hoc test). (b) In the systemic study, mean von Frey hair force flinching response thresholds (FRT, g ± sd) were determined in all groups and gabapentin (GP, 75 mg/kg, n = 8) was administered i.p. The responses were measured post treatment at 1 and 2 hours. (Statistical significance of differences in FRT were between saline control (SAL, n = 15) vs streptozotocin control (STZ, n = 8; ** P < 0.01) and from streptozotocin control (STZ, n = 8) vs gabapentin alone without streptozotocin pretreatment (GP, n = 8; * P < 0.05, ** P < 0.01), STZ + GP (n = 6) at 1 and 2 hours post drug treatment (* P < 0.05), ANOVA with Tukey’s post hoc analysis test). (GIF 204 kb) [file 210_2015_1145_Fig12_ESM.gif]

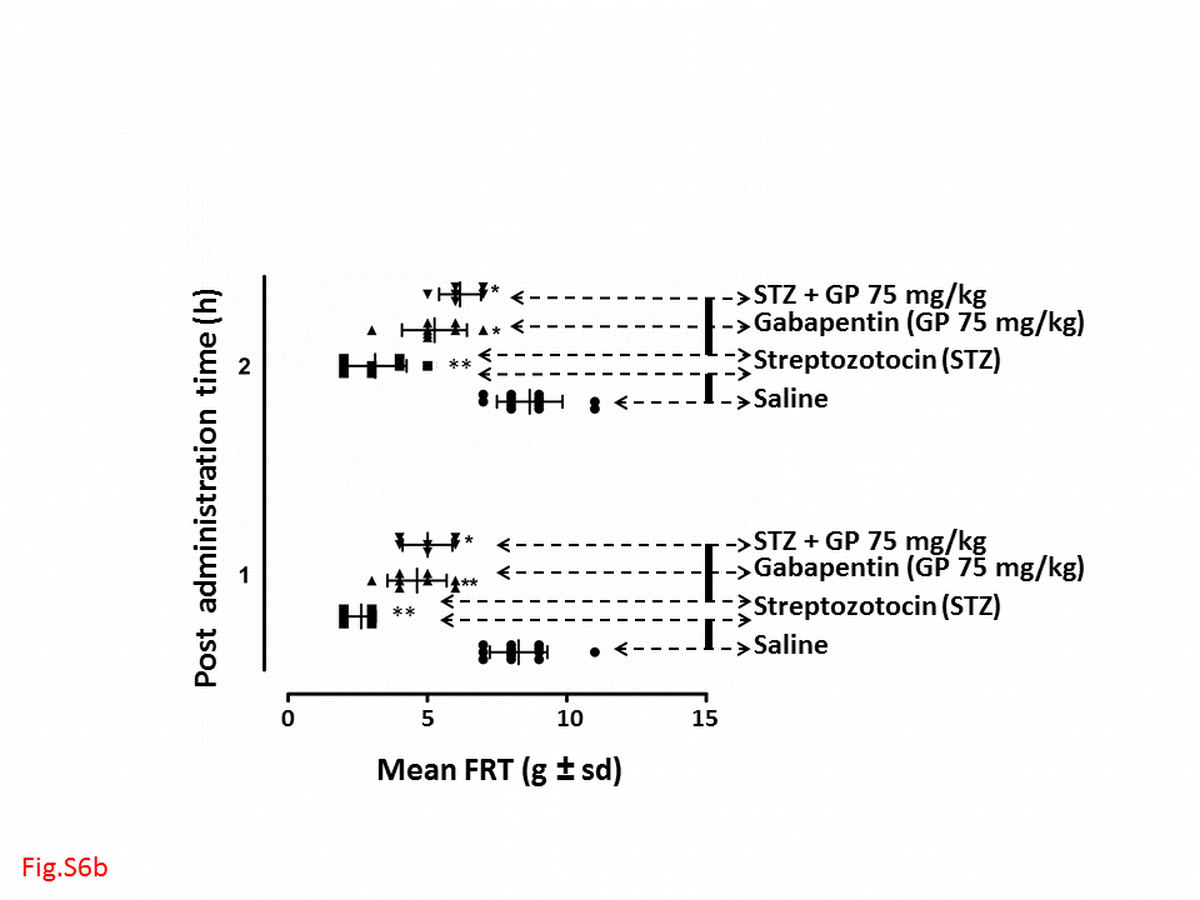

Supplement: Fig. 6 — The static anti-vulvodynia sffect of gabapentin gel (10 %) and systemic gabapentin (75 mg /kg i.p.) in streptozotocin-induced diabetic female rats. Scatter plots showing mean von Frey hair force flinching response thresholds (FRT, g ± sd) were determined in all groups (n = animal group numbers meeting the inclusion criteria are shown in brackets). (a) Control gabapentin gel alone without streptozotocin pretreatment (GG, n = 8), streptozotocin pretreatment with either control gel (STZ + CG, n = 13) or gabapentin gel (10 %) (STZ + GG, n = 14) were applied on the anogenital area including mons pubis of rats three times daily at the times shown and responses were measured 1 hour later. (Significance of differences in FRT between saline control (SAL, n = 15) vs streptozotocin control (STZ, n = 8; ** P < 0.01, *** P < 0.001) and between streptozotocin control (STZ, n = 8) vs gabapentin gel alone without streptozotocin pretreatment (GG, n = 8; * P < 0.05, ** P < 0.01), streptozotocin pretreatment followed by control gel i.e. STZ + CG (n = 13; P > 0.05) and streptozotocin pretreatment followed by gabapentin gel i.e. STZ + GG (n = 14; * P < 0.05) application, ANOVA followed by Tukey’s post hoc test). (b) In the systemic study, mean von Frey hair force flinching response thresholds (FRT, g ± sd) were determined in all groups and gabapentin (GP, 75 mg/kg, n = 8) was administered i.p. The responses were measured post treatment at 1 and 2 hours. (Statistical significance of differences in FRT were between saline control (SAL, n = 15) vs streptozotocin control (STZ, n = 8; ** P < 0.01) and from streptozotocin control (STZ, n = 8) vs gabapentin alone without streptozotocin pretreatment (GP, n = 8; * P < 0.05, ** P < 0.01), STZ + GP (n = 6) at 1 and 2 hours post drug treatment (* P < 0.05), ANOVA with Tukey’s post hoc analysis test). (GIF 204 kb) [file 210_2015_1145_Fig13_ESM.gif]

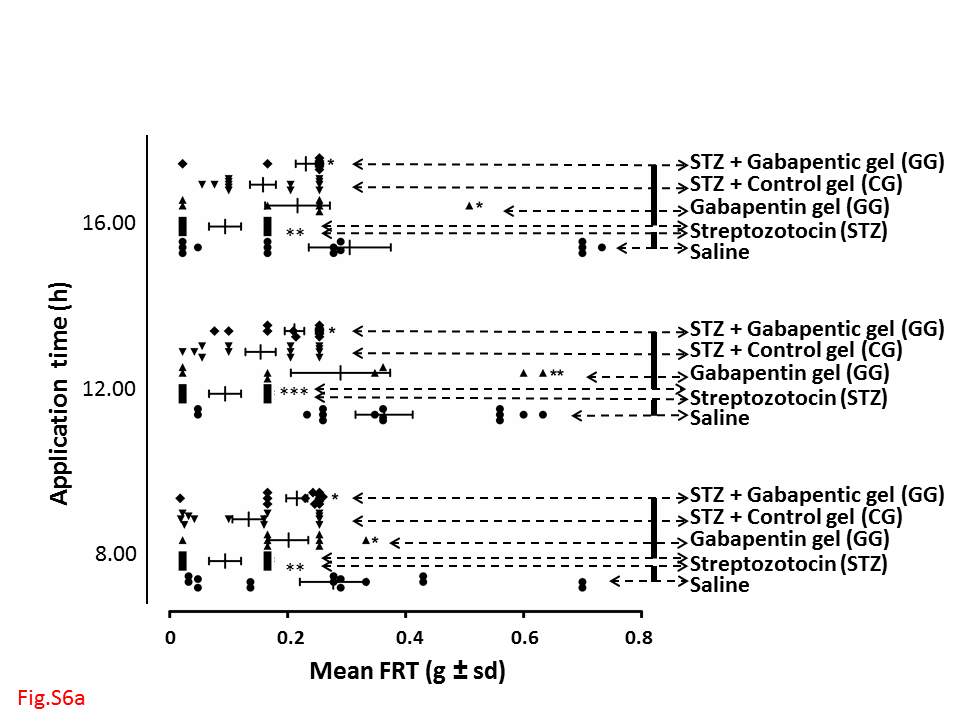

Supplement: Supplementary file 11 — High resolution image (TIFF 92 kb) [file 210_2015_1145_MOESM5_ESM.tif]

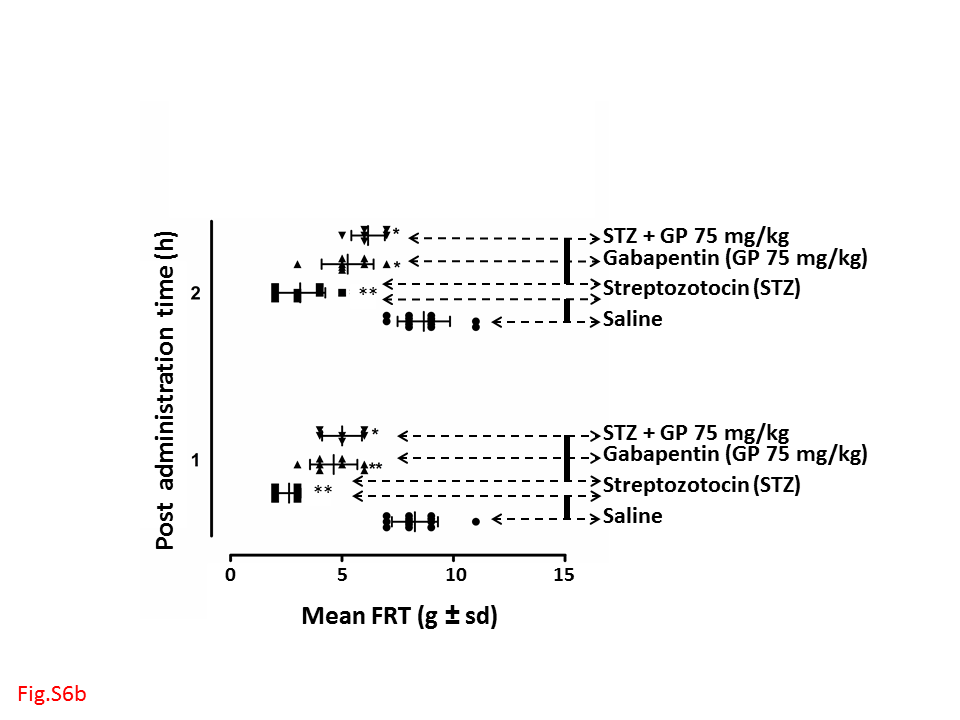

Supplement: Supplementary file 12 — High resolution image (TIFF 55 kb) [file 210_2015_1145_MOESM6_ESM.tif]

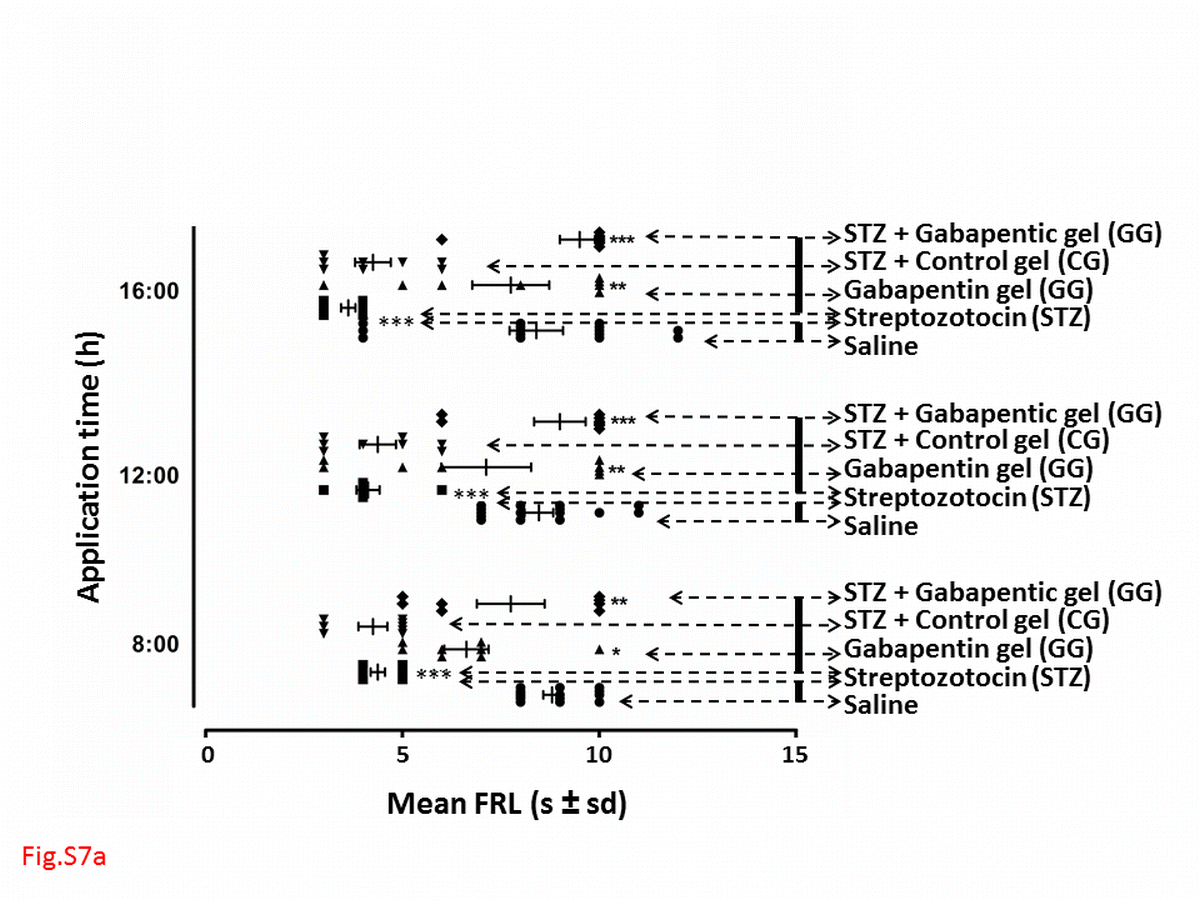

Supplement: Fig. 7 — The dynamic anti-vulvodynia effect of gabapentin gel 10 % and systemic gabapentin (75 mg /kg i.p.) in streptozotocin-induced female diabetic rats. Scatter plots showing mean flinching response latencies (FRL, s ± sd) of the anogenital area (vulva) in response to light brushing were determined in all groups (n = animal group numbers meeting the inclucion criteria are shown in brackets). (a) Control gabapentin gel alone without streptozotocin pretreatment (GG, n = 8), streptozotocin pretreatment followed by gabapentin gel (10 %) (STZ + GG, n = 8) or streptozotocin pretreatment followed by contron gel (STZ + CG, n = 8) were applied to the anogenital area (vulva) of rats three time daily at the times shown and FRL was measured 1 hour later. (Significance of differences in FRL were saline control (SAL, n = 15) vs streptozotocin control (STZ, n = 8; *** P < 0.001), gabapentin gel without streptozotocin pretreatment (n = 8) vs streptozotocin control STZ (n = 8; * P < 0.05, ** P < 0.01) and streptozotocin pretreatment followed by gabapentin gel (STZ + GG, n = 8) treatments in the morning (** P < 0.01), noon and afternoon (*** P < 0.001), ANOVA with post hoc Tukey’s test). (b) In the systemic study, the flinching response latencies (FRL) were determined in each group using the same protocol as for gabapentin gel and was plotted against post administration time (hours). The FRL was measured at 1 and 2 hours post gabapentin (GP, 75 mg /kg i.p.). ANOVA with Tukey’s post hoc analysis revealed significances in FRL between either the control gabapentin alone without streptozotocin pretreatment group (GP, 75 mg/kg, n = 8; * P < 0.05, ** P < 0.01) or saline controls (SAL, n = 15) vs streptozotocin controls (STZ, n = 8; ** P < 0.01). There were subsequent increases in FRL caused by gabapentin following streptozotocin pretreatment (STZ + GP, n = 6) at 1 hour (* P < 0.05) and 2 hours (* P < 0.05). (GIF 206 kb) [file 210_2015_1145_Fig14_ESM.gif]

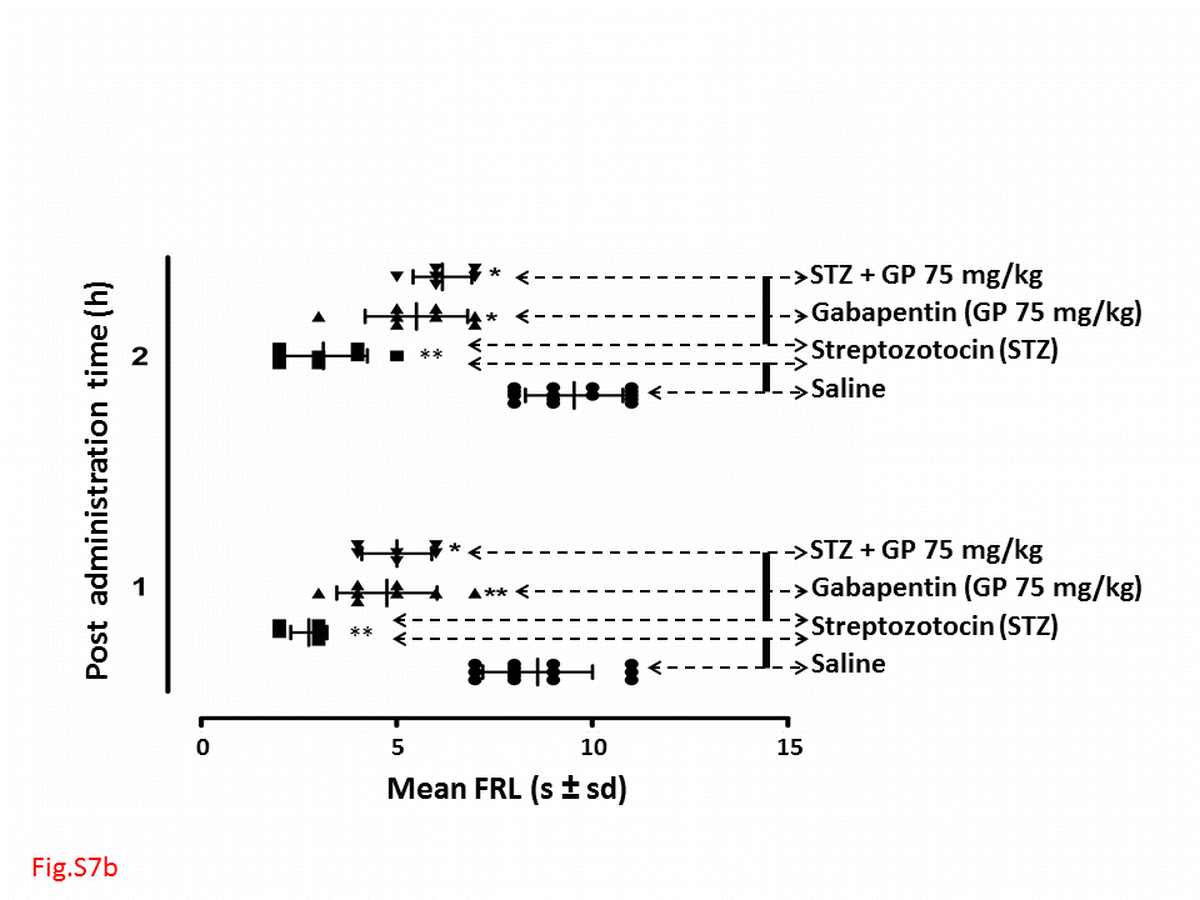

Supplement: Fig. 7 — The dynamic anti-vulvodynia effect of gabapentin gel 10 % and systemic gabapentin (75 mg /kg i.p.) in streptozotocin-induced female diabetic rats. Scatter plots showing mean flinching response latencies (FRL, s ± sd) of the anogenital area (vulva) in response to light brushing were determined in all groups (n = animal group numbers meeting the inclucion criteria are shown in brackets). (a) Control gabapentin gel alone without streptozotocin pretreatment (GG, n = 8), streptozotocin pretreatment followed by gabapentin gel (10 %) (STZ + GG, n = 8) or streptozotocin pretreatment followed by contron gel (STZ + CG, n = 8) were applied to the anogenital area (vulva) of rats three time daily at the times shown and FRL was measured 1 hour later. (Significance of differences in FRL were saline control (SAL, n = 15) vs streptozotocin control (STZ, n = 8; *** P < 0.001), gabapentin gel without streptozotocin pretreatment (n = 8) vs streptozotocin control STZ (n = 8; * P < 0.05, ** P < 0.01) and streptozotocin pretreatment followed by gabapentin gel (STZ + GG, n = 8) treatments in the morning (** P < 0.01), noon and afternoon (*** P < 0.001), ANOVA with post hoc Tukey’s test). (b) In the systemic study, the flinching response latencies (FRL) were determined in each group using the same protocol as for gabapentin gel and was plotted against post administration time (hours). The FRL was measured at 1 and 2 hours post gabapentin (GP, 75 mg /kg i.p.). ANOVA with Tukey’s post hoc analysis revealed significances in FRL between either the control gabapentin alone without streptozotocin pretreatment group (GP, 75 mg/kg, n = 8; * P < 0.05, ** P < 0.01) or saline controls (SAL, n = 15) vs streptozotocin controls (STZ, n = 8; ** P < 0.01). There were subsequent increases in FRL caused by gabapentin following streptozotocin pretreatment (STZ + GP, n = 6) at 1 hour (* P < 0.05) and 2 hours (* P < 0.05). (GIF 206 kb) [file 210_2015_1145_Fig15_ESM.gif]

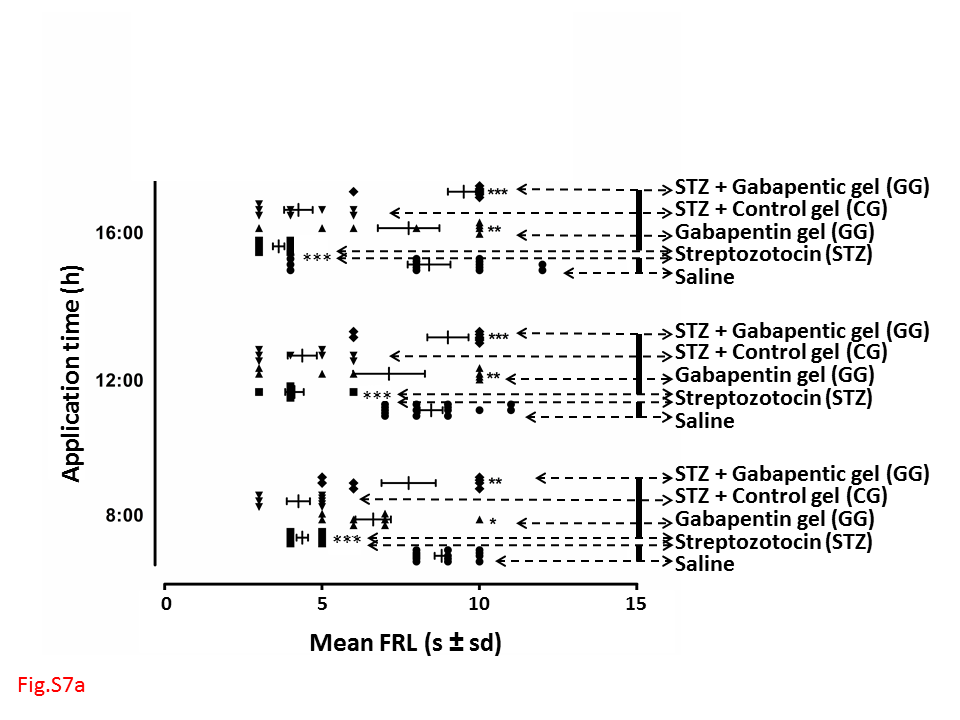

Supplement: Supplementary file 15 — High resolution image (TIFF 92 kb) [file 210_2015_1145_MOESM7_ESM.tif]

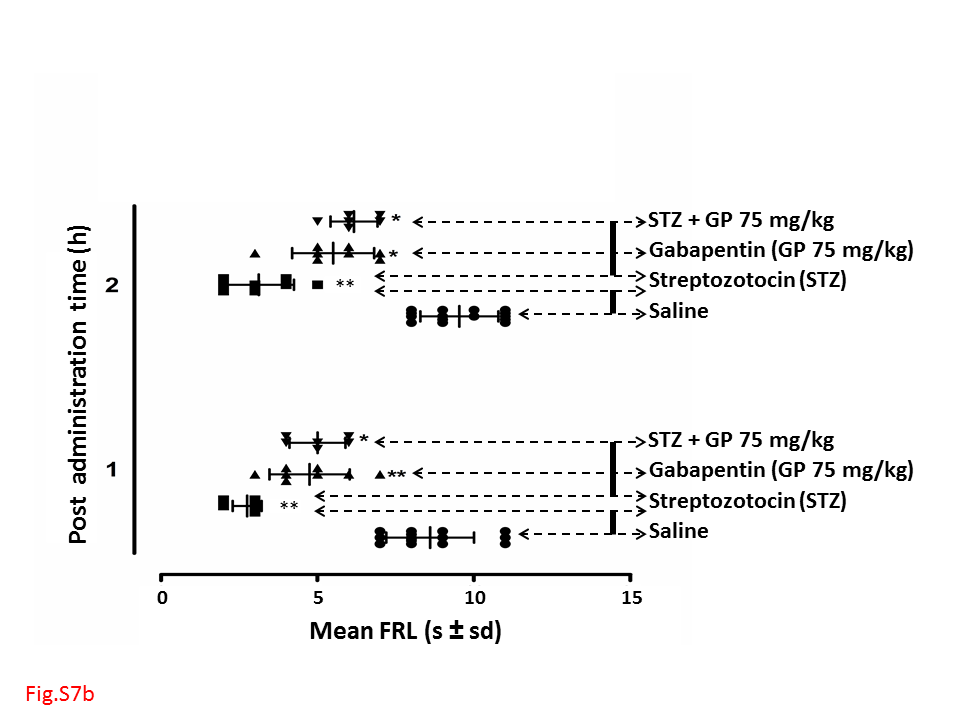

Supplement: Supplementary file 16 — High resolution image (TIFF 62 kb) [file 210_2015_1145_MOESM8_ESM.tif]
